# Synergistic Cu Doping and Yb Alloying Enhance Thermoelectric Performance of p‑Type Mg_1.8_Zn_1.2_Sb_2_‑Based Material toward High-Efficiency All-Mg_3_Sb_2_ Devices

**DOI:** 10.1021/acsami.5c22459

**Published:** 2026-01-30

**Authors:** Krushna K. Raut, Raju Chetty, Jayachandran Babu, Andrei Novitskii, Vikrant Trivedi, Takao Mori

**Affiliations:** † Graduate School of Pure and Applied Sciences, University of Tsukuba, 1-1-1 Tennodai, Tsukuba, Ibaraki 305-8577, Japan; ‡ Research Center for Materials Nanoarchitectonics (MANA), National Institute for Materials Science (NIMS), 1-1 Namiki, Tsukuba, Ibaraki 305-0044, Japan

**Keywords:** Seebeck coefficient, electrical conductivity, figure of merit, power generation, TE module, all Mg_3_Sb_2_-based device, maximum conversion efficiency

## Abstract

*AB*_2_Sb_2_-type Zintl phases, particularly Mg_3_Sb_2_-based materials, have recently garnered significant attention owing to their earth-abundant, nontoxic constituents and excellent *n*-type thermoelectric (TE) performance in the medium temperature range. However, achieving high-performance all Mg_3_Sb_2_-based TE devices remains difficult due to the lack of a compatible and efficient *p*-type counterpart. Herein, a combined approach of Cu doping and Yb alloying is employed to synergistically optimize the carrier concentration, carrier mobility, and reduce lattice thermal conductivity, thereby achieving a high-performance *p*-type Mg_3_Sb_2_ TE material. Consequently, a high *zT* value of 0.95 is obtained for the optimized composition Mg_1.18_Cu_0.02_Zn_1.2_Yb_0.6_Sb_2_ at 673 K. To demonstrate practical applicability, a fully compatible Mg_3_Sb_2_-based TE device is fabricated using cupronickel as a common diffusion barrier for both *p-* and *n*-type legs, ensuring excellent interfacial stability and device reliability. A crack-free interface with a specific contact resistivity of ρ_c_ ∼ 2.76 μΩcm^2^ is achieved. The single-leg p-type device exhibited a maximum efficiency, η_max_ ∼ 5.5%, while for a two-pair TE device, η_max_ ∼ 9.2% at a temperature difference (Δ*T*) of 374 K is realized. These findings demonstrate the potential of compositional engineering for developing efficient *p*-type Mg_3_Sb_2_ materials and fully integrated TE devices for waste heat recovery.

## Graphical abstract

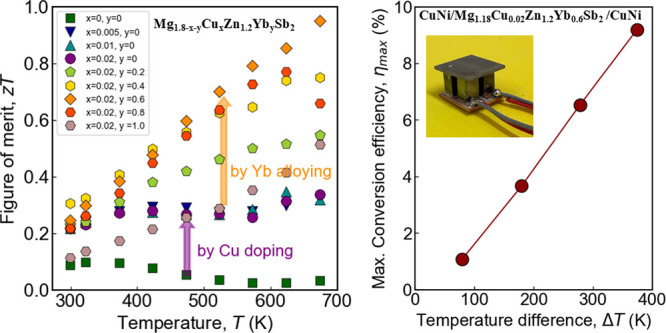

## Introduction

The accelerating global demand for energy and the urgent need for environmental sustainability have intensified the focus on technologies that efficiently harvest and utilize waste energy. Thermoelectric (TE) materials are particularly promising in this regard because of their unique ability to directly convert heat into electrical energy without moving parts, offering clean and reliable energy conversion.^1^ Their broad application spectrum spans from waste heat recovery in industrial and automotive sectors to powering remote sensors and portable electronics.^2,3^ The effectiveness of TE materials is governed by the dimensionless figure of merit, zT=α2σTκtot , where α is the Seebeck coefficient, σ is the electrical conductivity, *T* is the absolute temperature, and κ_tot_ is the total thermal conductivity.^4^ Achieving a high *zT* requires a balance between electronic and thermal transport, presenting long-standing material design challenges for efficient device operation.

Among emerging TE materials, Mg_3_(Sb,Bi)_2_-based Zintl phases are attractive owing to their earth-abundant, nontoxic constituents and high performance over a wide temperature range.^5–19^ Recently, *n*-type Mg_3_(Sb,Bi)_2_-based compounds have emerged at the forefront of TE research, owing to their exceptional figure of merit (*zT*), finely engineered interface layers, superior mechanical robustness, and outstanding device performance.^5–8^ These *n*-type Mg_3_(Sb,Bi)_2_ materials are commonly coupled with *p*-type MgAgSb,^8,10,20–23^ Bi_2_Te_3_,^24–26^ CoSb_3_,^27^ CdSb,^28^ and GeTe-based^29^ counterparts to construct single-pair or multipair TE devices. However, the *p*-type counterparts pose critical challenges owing to their mechanical incompatibility and the incorporation of toxic or high-cost elements, which limit their large-scale applicability.^9–12,14–19^ Therefore, developing *p*-type materials with improved mechanical compatibility that are composed of environmentally benign materials is of great importance. In this regard, *p*-type Mg_3_Sb_2_-based materials have been emerging as promising candidates due to their earth-abundant constituents, favorable mechanical properties, and potential for achieving high TE performance.^30–33^ The realization of all-Mg_3_Sb_2_ TE devices comprising both *p*-type and *n*-type legs with harmonized structural and thermal characteristics represents a critical milestone toward practical device implementation. Utilizing materials from the same chemical and crystallographic family ensures compatibility in thermal expansion coefficients, mechanical robustness, and chemical stability during thermal cycling, thereby minimizing interfacial degradation and stress-induced failure. Moreover, the module architecture as well as standardization also plays a crucial role in determining the efficiency. Recent studies have emphasized that optimized module architecture and standardized evaluation are essential for translating high-performance TE materials into practical devices.^34^

Recently, Liang et al.^30^ reported that double substitution at the Mg sites in *p*-type Mg_3_Sb_2_ effectively optimizes the carrier concentration and enhances phonon scattering, leading to improved TE performance and enhanced device stability due to the closely matched thermal expansion coefficients between the *p*- and *n*-type legs. A unicouple TE device fabricated from these materials exhibited a maximum conversion efficiency (η_max_) of ∼5.5% at a hot-side temperature of 573 K. Meanwhile, Jiang et al.^31^ reported an all-Mg_3_Sb_2_-based eight-pair TE device with matched thermomechanical properties, optimized joining, and diffusion barriers, achieving η_max_ of 7.5% at a temperature difference (Δ*T*) of 380 K and maintaining excellent stability over 150 thermal cycles. Similarly, Hu et al.^32^ highlighted the critical role of defect-induced electron scattering for *p*-type Mg_3_Sb_2_-based materials and device-level innovations, such as thermal expansion, matched diffusion barrier layers, and transient liquid phase (TLP) bonding to realize a η_max_ of 8.3% at a Δ*T* ≈ 430 K with good thermal stability. Moreover, Lei et al.^33^ proposed a band engineering strategy that involves selectively alloying Zn at the *Mg_2_* sites and Yb at the *Mg*_1_ sites in Mg_3_Sb_2_ and later Li doping for carrier optimization. This approach effectively increases valence band degeneracy while simultaneously reducing the band effective mass with coalloying, resulting in a significant enhancement of the power factor (PF) and achieving a record *zT* ∼ 1.4 for Mg_0.897_Li_0.003_Zn_1.4_Yb_0.7_Sb_2_ at 800 K.

Inspired by these studies on optimization of carrier concentration by doping and band engineering via alloying, a *p*-type composition of Mg_1.8_Zn_1.2_Sb_2_ was selected as the pristine TE material. Based on the previous study,^30^ the effect of Zn alloying on the Mg site of Mg_3_Sb_2_ and its beneficial TE properties, we selected Mg_1.8_Zn_1.2_Sb_2_ as a pristine compound to explore the influence of additional compositional modification (i.e., Cu doping and Yb alloying effect). In the present work, the carrier concentration of the pristine sample is optimized by the Cu doping on the Mg site, leading to an increase of PF from 2.5 μWcm^–1^K^–2^ for the pristine sample to 7.1 μWcm^–1^K^–2^ for Mg_1.78_Cu_0.02_Zn_1.2_Sb_2_ at 300 K. As a result, a maximum *zT* of ∼0.35 at 423 K for Mg_1.78_Cu_0.02_Zn_1.2_Sb_2_ was achieved. However, the elevated lattice thermal conductivity combined with bipolar conduction significantly reduces *zT*. Subsequently, Yb was incorporated at the *Mg1* site to enhance electronic transport further and suppress the lattice thermal conductivity to realize higher *zT*. Consequently, the optimized chemical composition Mg_1.18_Cu_0.02_Yb_0.6_Zn_1.2_Sb_2_ exhibits favorable electronic transport due to the increased carrier mobility and significant suppression of lattice thermal conductivity due to the increased phonon scattering by the alloying effect. The combined effect of Cu doping and Yb alloying led to an increase in PF of 10 μWcm^–1^K^–2^ and *zT* of ∼0.95 at 673 K for the Mg_1.18_Cu_0.02_Yb_0.6_Zn_1.2_Sb_2_.

Following the optimization of the bulk TE material properties, Cu–Ni alloy (cupronickel)-based contact interface layers for *p*-type Mg_1.18_Cu_0.02_Yb_0.6_Zn_1.2_Sb_2_ were developed to ensure good thermomechanical compatibility with the *n*-type counterpart Mg_3_(Sb,Bi)_2_-based TE materials. A good electrical contact with a low specific contact resistivity (ρ_c_∼ 2.76 μΩcm^2^) is realized at the interface between the TE material and contact interface layer, which results in a single TE leg η_max_ ∼ 5.5% at Δ*T* = 365 K. By using the same contact interface layer for both the *p*-type Mg_1.18_Cu_0.02_Yb_0.6_Zn_1.2_Sb_2_ and *n*-type Mg_3_(Sb,Bi)_2_ ^8^ materials, a two-pair TE device is fabricated. The two-pair TE device exhibits a η_max_ ∼ 9.2% at Δ*T* = 374 K. This study integrates materials optimization, from carrier and phonon engineering to device level assembly, to advance Mg_3_Sb_2_-based TE toward practical, high-performance energy conversion technologies.

## Experimental Details

### Materials Synthesis

High-purity elements Mg (99.999%, 5N plus), Sb (99.999%, 5N plus), Zn (99.995%, Kojundo Chemicals), Yb (99.9%, Thermo Fisher Scientific), and Cu (99.9%, Kojundo Chemicals) were weighed with the nominal chemical compositions of Mg_1.8–*x*_Cu_*x*_Zn_1.2_Sb_2_ (*x* = 0, 0.005, 0.01, 0.02) and Mg_1.78‑*y*_Cu_0.02_Yb_*y*_Zn_1.2_Sb_2_ (*y* = 0.2, 0.4, 0.6, 0.8, 1.0). The initial mixtures were loaded into a stainless-steel ball milling (BM) jar. Weighing and loading of the elements were carried out inside an argon-filled glovebox, maintaining an oxygen level below 1.0 ppm. Subsequently, the powders were continuously milled for 10 h using a SPEX 8000 D mill/mixer. The resulting ball milled powders were then transferred to a graphite die (inner diameter: 10 mm) and immediately consolidated by spark plasma sintering (SPS-1080 system, SPS SYNTEX INC) at 923 K for 5 min under an applied pressure of ∼60 MPa.

## Materials Characterization

Phase analysis and lattice constants were obtained by using powder X-ray diffraction (XRD; MiniFlex, Rigaku, Japan). The XRD patterns were collected at room temperature within a 2θ range of 10–90°, employing a step size of 0.02 °/min and Cu*K*_α_ radiation (λ_Cu_ = 1.5406 Å). Rietveld refinement was performed with the FullProf software suite.^35^ The morphology and elemental composition of the sintered samples were analyzed by using field emission scanning electron microscopy (FESEM; Hitachi SU8230, Hitachi High-Tech, Japan) equipped with an energy dispersive X-ray spectroscopy detector (EDS; X-Max^N^ EDS detector, Horiba Scientific, Japan). Sintered compacts were sectioned into rectangular bars for simultaneous evaluation of the Seebeck coefficient (α) and electrical conductivity (σ), while disk-shaped samples (10 mm diameter, ∼1.5 mm thickness) were utilized for thermal diffusivity measurements. α and σ were determined by the four-probe technique using a commercial system (ZEM-3, Advance Riko, Japan). The total thermal conductivity (κ_tot_) was calculated from κ_tot_ = *d*ρ*C*_*p*_, where *d* is the thermal diffusivity, *C*_*p*_ is the specific heat capacity, and ρ is the bulk density. *d* was measured via the laser flash technique (LFA 467 HyperFlash, Netzsch, Germany). The density ρ was obtained using Archimedes’ method. The temperature-dependent *C*_*p*_ was calculated using the polynomial expression developed by Agne et al.,^36^ which accurately models the heat capacity of Mg_3_Sb_2_ and its alloys with a wide range of temperatures. The details of the calculation are given in the SI Section. The lattice contribution to thermal conductivity (κ_lat_) was derived by subtracting the electronic component (κ_ele_) from κ_tot_. The electronic part of thermal conductivity (κ_ele_ = *L*σ*T*) is calculated from the Weidemann-Franz relation using the temperature-dependent Lorenz number, *L*(*T*), estimated from the SPB model (see the SI Section for details). Electrical resistivity and Hall effect measurements were performed at room temperature using a standard five-probe setup integrated into a physical property measurement system (PPMS-9T, Quantum Design Inc., USA). Longitudinal and transverse sound velocities were measured by a sing-around ultrasonic velocity measuring instrument (UVM-2, Ultrasonic Engineering Co., Japan) at room temperature. The estimated uncertainties were 6% for α, 8% for σ, 11% for κ_tot_, 8–10% for the Hall data, and 16% for the overall *zT*.^37^ Error bars are omitted in certain figures for the sake of clarity.

## Fabrication of Electrical Contacts and Prototype Devices

An electrical contact development study was carried out on the optimized Mg_1.8–*y*_Cu_0.02_Yb_*y*_Zn_1.2_Sb_2_ composition. Cupronickel (Cu_70_Ni_30_, Nilaco Corporation) powder was used as a potential electrical contact. Solid-state diffusion-based techniques, such as one-step sintering, were initially tried for the single TE leg fabrication. The one-step sintering was carried out under the same SPS conditions as those used for material densification, which resulted in cracking and excessive melting. Thus, the two-step sintering was carried out at a temperature (773 K) lower than the TE material’s SPS temperature, followed by a similar procedure as previously reported for *n*-type.^38^ The fabricated TE discs were diced to cuboid-shaped TE legs of ∼3 × 3 × 5 mm^3^ size for characterization. Two-pair TE devices were fabricated by combining the *p*-type Mg_1.18_Cu_0.02_Yb_0.6_Zn_1.2_Sb_2_ with an optimized *n*-type Mg_3_Sb_1.5_Bi_0.5_-based material reported previously.^8^ Among the various electrical contacts studied in this work, CuNi was chosen for the *p*-type Mg_1.18_Cu_0.02_Yb_0.6_Zn_1.2_Sb_2_ material. CuNi was previously reported as an effective contact for the *n*-type Mg_3_Sb_1.5_Bi_0.5_ material too.^38^ Hence, a fully Mg_3_Sb_2_-based device with the same contact layers was demonstrated. The TE legs of ∼5 mm height and ∼3 × 3 mm^2^ cross-sectional area for both *p*-type and *n*-type were assembled on a 10 × 10 mm^2^ Cu substrate with polymer insulation and Cu patterns on the cold side and directly bonded alumina substrates on the hot side using commercial In–Ga solder paste (Signa Aldrich). Four lead wires were soldered to the terminals for device performance characterization.

## Characterization of TE Legs and Prototype Device

Electrical specific contact resistivity (ρ_c_) at room temperature was estimated from the resistance jump (Δ*R*) across the contact region, measured by a linear resistance profiling system (Mottinai Energy, Japan), using the equation: ρ_c_ = Δ*R*·*A*_c_, where *A*_c_ is the TE leg cross-sectional area. The microstructural analysis across the contact region was performed using the same SEM-EDS system used for materials characterization. The power generation characteristics of single legs and the two-pair prototype device were estimated using commercial simulation software (COMSOL, Inc., Sweden ). The heat and electric transport were modeled using heat transfer and AC/DC modules using temperature-dependent TE material properties. The *V*-*I*, *P*-*I*, *Q*_out_-*I*, and η-*I* characteristics were calculated at different Δ*T*. Experimental performance testing of the single TE legs was conducted by using a commercial power generation evaluation system (MiniPEM, Advance Riko, Japan). The hot-side temperature varied from 373 to 673 K with a 100 K step, while the cold-side temperature was maintained at ∼298 K. The terminal voltage (*V*) was measured as a function of input electric current (*I*) from open-circuit (*V* = *V*_oc_, *I* = 0) to short-circuit (*V* = 0, *I* = *I*_sc_) conditions at each Δ*T*. The output power (*P*) was calculated using the equation, *P* = *VI*. The heat flow (*Q*_out_) through the TE legs/device was measured by using a flow calorimeter attached to the cold side. The power conversion efficiency (η) was calculated using the relation, η=PP+Qout .^39^

## Results and Discussion

### Enhancing *zT* by Tuning Carrier Concentration via Cu Doping

Cu, having one fewer valence electron than Mg, can serve as a potential *p*-type dopant in the *AB*_2_Sb_2_ Zintl system. Previous studies have primarily focused on alkali elements such as Li, Na, and K as effective *p*-type dopants.^13,16,41–43^ However, owing to its metallic character, Cu is expected to offer enhanced structural stability and improved microstructural control in addition to facilitating carrier concentration optimization.

Figure 1 shows the powder XRD patterns of all of the samples Mg_1.8–*x*_Cu_*x*_Zn_1.2_Sb_2_ (*x* = 0.005, 0.01, and 0.02). The powder XRD of sintered pellets confirms the main phase corresponds to a layered Mn_2_O_3_-type crystal structure (space group P3®m1 ). A peak shift toward a higher 2θ angle in the XRD pattern is observed upon Cu doping, confirming the decrease of the lattice spacing. Further, the lattice parameters of Cu-doped samples are evaluated using the Rietveld refinement by substituting Cu at the *Mg1* site of the Mg_3_Sb_2_ structure. The lattice parameters decrease with an increase of Cu content (Table S1). This is due to the smaller atomic radius of Cu (140 pm) than that of Mg (160 pm).^44^ Microstructure analysis indicates that the samples are highly densely packed, suggesting a well-compacted and uniform microstructure (see SI Figure S1). Moreover, the EDS point analysis was carried out on the optimum Yb-doped sample Mg_1.78_Cu_0.02_Zn_1.2_Sb_2_ to confirm the actual composition. The EDS analysis revealed that the actual chemical composition is in good agreement with the nominal composition, with a slight deviation, which is within the experimental error limits (Table S2).

**Figure 1:**
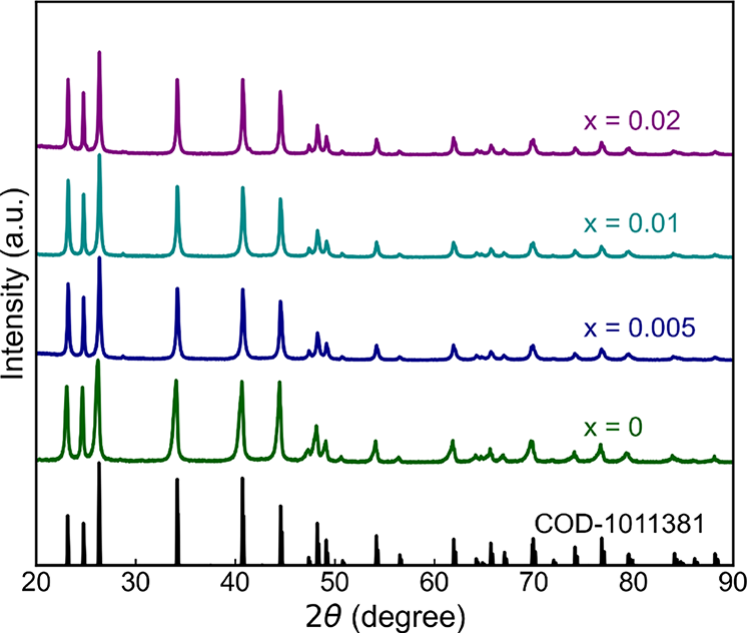
Powder XRD patterns of Mg_1.8–*x*_Cu_*x*_Zn_1.2_Sb_2_ (*x* = 0, 0.005, 0.01, and 0.02). The main diffraction peaks were matched with corresponding peaks of Mg_1.8_Zn_1.2_Sb_2_ (COD-1011381).^40^

Figure 2 shows the TE properties of Mg_1.8–*x*_Cu_*x*_Zn_1.2_Sb_2_ (*x* = 0, 0.005, 0.01, and 0.02). The electrical conductivity decreases with the Cu substitution on the Mg site (Figure 2a). At 300 K, the σ value increases significantly from ∼32 S cm^–1^ for the pristine sample to ∼200 S cm^–1^ for Mg_1.79_Cu_0.01_Zn_1.2_Sb_2_. Nearly a 6-fold increase in electrical conductivity can be primarily attributed to the enhanced carrier concentration (*n*) resulting from Cu substitution (Figure 2c). For Mg_1.78_Cu_0.02_Zn_1.2_Sb_2_, the σ slightly decreases to ∼192 S cm^–1^ at 300 K attributable to a reduction in carrier concentration, possibly due to the formation of secondary phases at Cu content above 0.01, although no such phases were detected by XRD. The impact of Cu on Hall mobility (μ) is negligible (Figure 2c), suggesting that Cu doping does not significantly alter the electronic band structure. The pristine sample exhibits semiconducting behavior in σ with temperature, whereas Cu-doped samples show a transition from metallic-like to semiconducting behavior around 475 K, corresponding to intrinsic conduction onset. Above 475 K, σ for all Cu-doped samples converges, attributed to carrier concentration saturation beyond this temperature; further increases in extrinsic carriers are limited or inhibited. The positive α in all samples confirms holes are the majority carriers (Figure 2b). At 300 K, the Seebeck coefficient decreases from ∼278 μV K^–1^ for the pristine sample to ∼174 μV K^–1^ for the Mg_1.79_Cu_0.01_Zn_1.2_Sb_2_, indicating an increase in carrier concentration (*n*) induced by Cu substitution at the Mg site (Figure 2c). A slight increase in α to ∼191 μV K^–1^ is observed for Mg_1.78_Cu_0.02_Zn_1.2_Sb_2_, consistent with its reduced carrier concentration. For the pristine sample, α decreases with temperature due to intrinsic conduction onset near room temperature, while Cu doping shifts this onset to higher temperatures (423 K). These variations in α and σ collectively govern the PF (α^2^σ), as shown in Figure 2d. The improved balance of carrier concentration and Seebeck coefficient enhances PF from ∼2.5 μWcm^–1^K^–2^ for the pristine sample to ∼7.1 μWcm^–1^K^–2^ for Mg_1.78_Cu_0.02_Zn_1.2_Sb_2_ at 300 K. Although lower than some previous reports, Cu substitution results in a 3-fold PF increase relative to undoped Mg_1.8_Zn_1.2_Sb_2_.^18,30,41^ The total thermal conductivity (κ_tot_) as a function of temperature for all of the samples is shown in Figure 2e. The κ_tot_ slightly increases with the Cu doping for the sample *x* = 0.005, while it decreases slightly with a higher Cu content. The increase in κ_tot_ is attributed to increase in κ_ele_, the carrier contribution to thermal conductivity. At 300 K, the κ_tot_values are in the range of 0.88–0.93 Wm^–1^K^–1^ for all the samples, indicating that there is no significant influence on κ_tot_ with Cu doping. All of the samples showed bipolar thermal conductivity, which resulted from the onset of intrinsic conduction. The temperature-dependent *zT* for all the samples is shown in Figure 2f. The combined effect of high α^2^σ and low κ_tot_ led to the maximum *zT* of 0.35 at 423 K for Mg_1.78_Cu_0.02_Zn_1.2_Sb_2_. Further increasing the Cu content resulted in a deterioration of *zT;* consequently, a Cu content of 0.02 was identified as the optimal doping level for enhancing *zT* via alloying.

**Figure 2:**
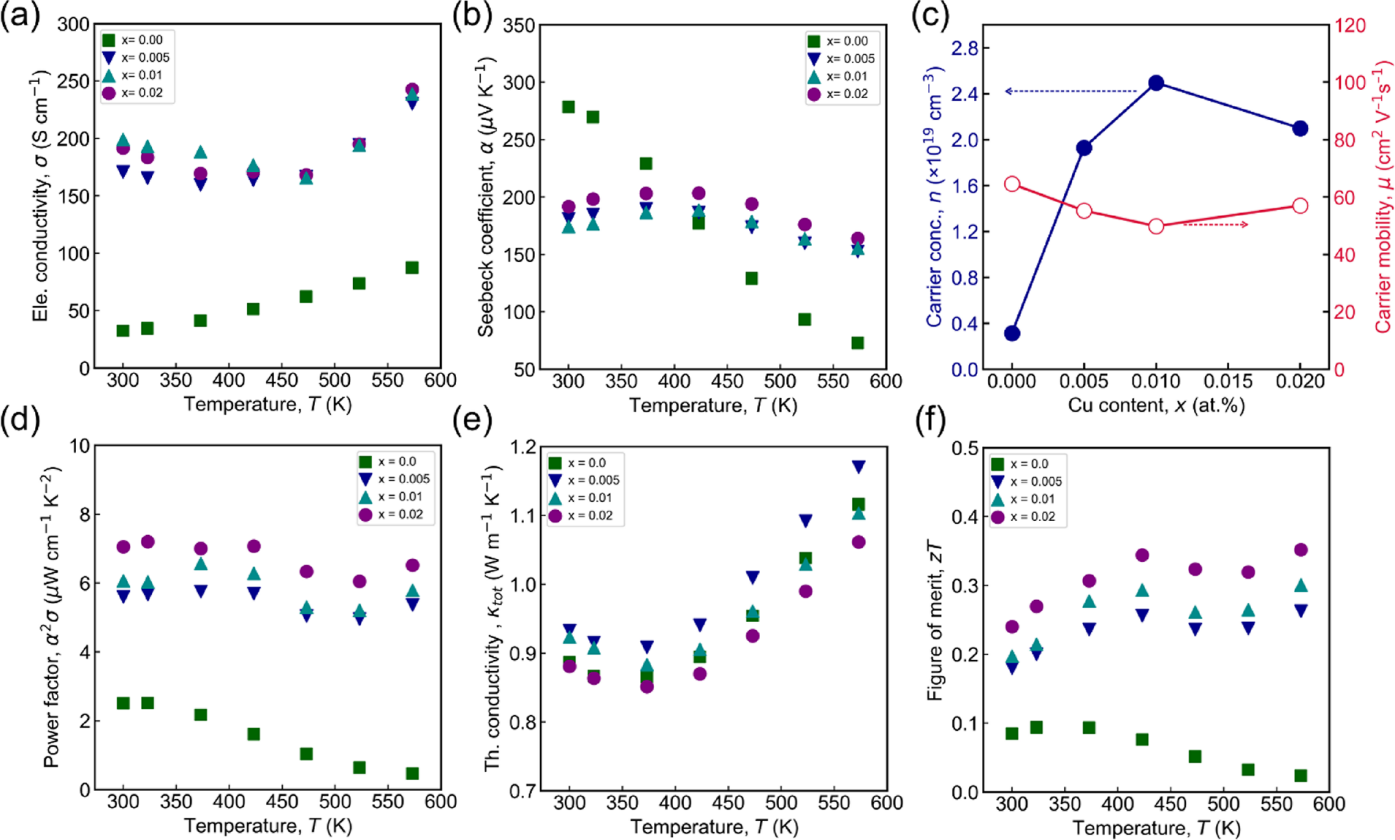
Thermoelectric properties of Mg_1.8–x_Cu_*x*_Zn_1.2_Sb_2_ (*x* = 0, 0.005, 0.01, and 0.02). Dependence of (a) σ, (b) α, (c) Dependence of *n* and μ on composition. (d) α^2^σ, (e) κ_tot_, and (f) *zT* on temperature.

### Enhancing *zT* by Yb Alloying

The beneficial impact of Yb alloying on TE properties in Mg-based Zintl compounds, including significant reductions in lattice thermal conductivity through enhanced mass and strain field fluctuations, as well as tuning of electronic band structure via band convergence and band shaping, has been firmly established in the recent literature.^16,30,32,33^ Building on these foundational insights, we systematically investigated Mg_1.78‑*y*_Yb_*y*_Cu_0.02_Zn_1.2_Sb_2_ (*y* = 0, 0.2, 0.4, 0.6, 0.8, and 1.0) and studied structural, microstructural, electrical, and thermal transport properties. The X-ray powder diffraction data of the sintered samples confirms the main phase corresponds to a layered Mn_2_O_3_-type crystal structure (space group P3®m1 ) as shown in Figure 3a. A trace of the Sb impurity peak is present in all the samples, which can be attributed to the Mg volatilization as reported previously.^45^ XRD study confirmed the successful incorporation of Yb through peak shifts toward lower angles and expansion of the lattice parameters consistent with substitutional alloying (see SI Figure S2). The Rietveld refinement results in Figure 3b show an increase in the lattice parameter upon Yb substitution, which is attributed to the replacement of Mg atoms (atomic radius of Mg is 160.2 pm) by larger Yb atoms (atomic radius of Yb is 193.9 pm) in the *Mg* *1*-site.^44^ SEM micrographs reveal two distinct microstructural features in the Yb-alloyed samples (see SI, Figure S3). First, void-like structures are observed, likely due to the vaporization or oxidation of Yb or Mg during sintering. Such void formation is a phenomenon commonly attributed to volatilization effects during high-temperature processing.^30^ Second, a notable reduction in grain size occurs in Yb-alloyed samples compared to those without Yb (see SI Figures S1 and S3). Li et al. also reported a reduction in grain size in alloyed *p*-type systems where Eu, Cd, Yb, and Ba were substituted at the *Mg* site.^16^ Reduction in grain size can play a significant role in lowering the lattice thermal conductivity by enhancing the scattering of phonons at grain boundaries.^46^

**Figure 3:**
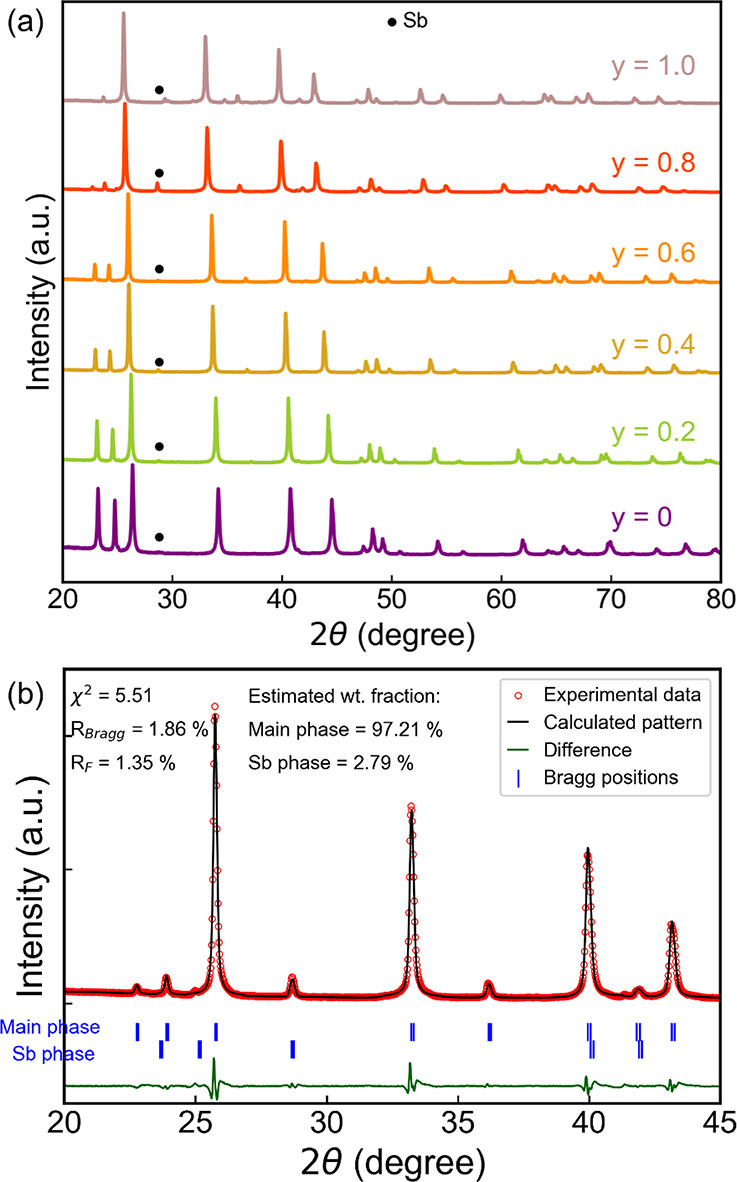
(a) Powder XRD patterns of Mg_1.78–*y*_Cu_0.02_Yb_*y*_Zn_1.2_Sb_2_ (*y* = 0, 0.2, 0.4, 0.6, 0.8, and 1.0). (b) XRD refinement details of the Mg_1.18_Cu_0.02_Yb_0.6_Zn_1.2_Sb_2_ composition.

Figure 4 shows the temperature-dependent TE properties of Mg_1.78‑*y*_Cu_0.02_Yb_*y*_Zn_1.2_Sb_2_ (*y* = 0, 0.2, 0.4, 0.6, 0.8, and 1.0). In Figure 4a, the electrical conductivity (σ) is plotted against the temperature, showing that a higher Yb content leads to increased σ values. Electrical conductivity is governed by the relation σ = *ne*μ, where *n* is the carrier concentration, *e* is the elementary charge, and μ is the carrier mobility. Mobility is further related to effective mass via μ = *e*τ/m*, where τ represents the scattering time, and m* is the effective mass. The carrier concentration at room temperature (Table 1) shows that the Yb substitution has a negligible influence on *n*. This is attributed to the isovalent nature of Yb and Mg, consistent with previous report.^30^ However, a significant increase in Hall mobility is observed with an increase in Yb content up to 0.6 in Mg_1.78‑*y*_Cu_0.02_Yb_*y*_Zn_1.2_Sb_2_ (*y* = 0, 0.2, 0.4, 0.6, 0.8, and 1.0) (see Table 1). For instance, mobility increases from 32 cm^2^V^–1^s^–1^ for Mg_1.58_Cu_0.02_Yb_0.2_Zn_1.2_Sb_2_ to 96 cm^2^V^–1^s^–1^ for Mg_1.18_Cu_0.02_Yb_0.6_Zn_1.2_Sb_2_ while the carrier concentration remains nearly constant (Table 1). Thus, the increase in electrical conductivity observed in the Yb-substituted samples is attributed to the higher mobility values (Table 1). Moreover, σ decreases with increasing temperature to ∼573 K, characteristic of degenerate semiconductors. Figure 4b shows the temperature dependence of the Seebeck coefficient. The value of α decreases with an increasing Yb content, directly reflecting the corresponding behavior of electrical conductivity. Although Yb substitution has a negligible effect on carrier concentration, a slight increase in *n* is observed. To assess the impact of Yb alloying, the band gap (*E*_g_) was calculated using the Goldsmid-Sharp relation, *E*_g_ = 2*e*|*S*_max_|*T*_max_, and found to be 0.13, 0.17, 0.25, 0.23, and 0.23 for *y* = 0, 0.2, 0.4, 0.6, and 0.8, respectively. It shows a gradual increase in band gap, which evidence that the bipolarity is successfully shifted toward a higher temperature than that of Mg_1.78_Cu_0.02_Zn_1.2_Sb_2_. The calculated band gap is comparable to the band gap predicted by DFT calculation by Shuai et al.^47^

**Figure 4:**
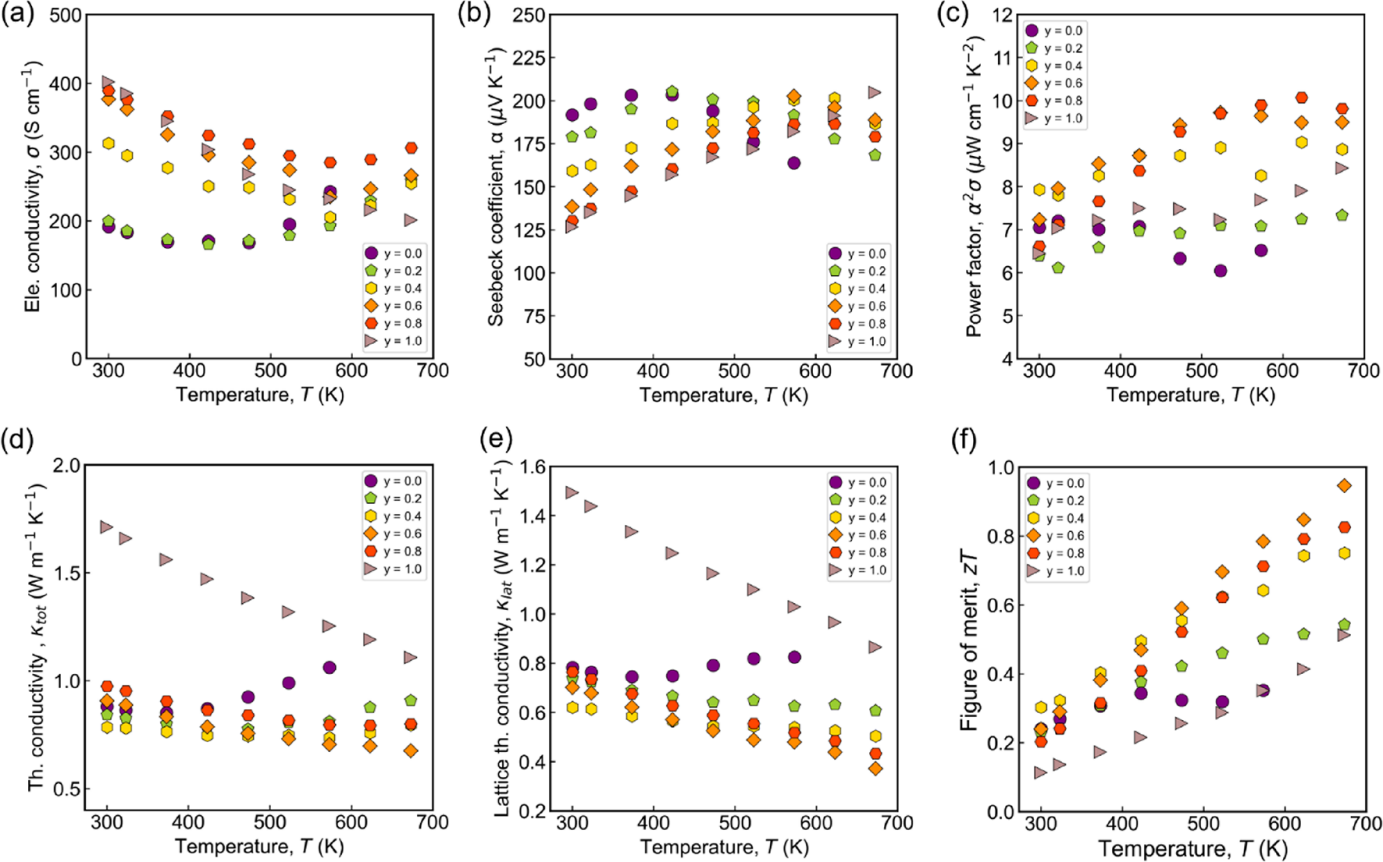
Temperature-dependent thermoelectric transport properties of (a) σ, (b) α, (c) α^2^σ, (d) κ_tot_, (e) κ_lat_, and (f) *zT* of Mg_1.78–*y*_Cu_0.02_Yb_*y*_Zn_1.2_Sb_2_ (*y* = 0, 0.2, 0.4, 0.6, 0.8, and 1.0).

**Table 1:** Carrier Concentration (*n*), Mobility (μ), and Effective Mass (m*_d_ */m*_*e*_) Values of Mg_1.78–*y*_Cu_0.02_Yb_*y*_Zn_1.2_Sb_2_ (*y* = 0, 0.2, 0.4, 0.6, and 0.8) at Room Temperature

sample nominal composition	*n*(cm^–3^)	μ (cm^2^V^–1^s^1^)	md*me
Mg_1.78_Cu_0.02_Zn_1.2_Sb_2_	2.10 × 10^19^	56.50	0.90
Mg_1.58_Cu_0.02_Yb_0.2_Zn_1.2_Sb_2_	3.27 × 10^19^	38.12	1.23
Mg_1.38_Cu_0.02_Yb_0.4_Zn_1.2_Sb_2_	2.50 × 10^19^	77.98	0.85
Mg_1.18_Cu_0.02_Yb_0.6_Zn_1.2_Sb_2_	2.44 × 10^19^	96.40	0.68
Mg_0.98_Cu_0.02_Yb_0.8_Zn_1.2_Sb_2_	3.17 × 10^19^	76.58	0.74

To elucidate the origin of enhanced electrical conductivity upon Yb substitution, the electrical transport behavior was analyzed using the single parabolic band (SPB) model to estimate the density of states effective mass (m*) (see SI Section for details).^48^ The calculated m* values exhibit a decreasing trend with an increasing Yb content up to *y* = 0.6, as summarized in Table 1. This trend is consistent with a previous study of Liang et al.^30^ and our calculated m* values show good agreement with the reported literature.^30,49,50^ The reduction in m* with Yb incorporation can be attributed to the band-shaping effect induced by Yb substitution reported previously, which modifies the valence band curvature and facilitates improved carrier mobility.^33^ The contribution of valence band structure in the *AB*_2_ *Sb*_2_ compound primarily arises from the *P*_*x,y*_ and *P*_*z*_ orbitals of the Sb, i.e., nondegenerate valence band minima 1 (VBM1) and doubly degenerate valence band minima 2 (VBM2). Previous studies have shown that coalloying with Zn and Yb shifts VBM2 closer to VBM1.^51^ This interaction leads to an enhanced valley degeneracy due to Zn and pronounced band sharpening due to Yb.^33,52^ However, Yb substitution induces band shaping that reduces the band effective mass (*m*_b_^*^). Consequently, the observed decrease in the overall m* can be attributed to the reduction in *m*_b_^*^ caused by Yb incorporation. The density-of-states effective mass is defined in terms of the band mass by accounting for the degeneracy and anisotropy of the electronic bands. For a semiconductor with multiple equivalent valleys (valley degeneracy, *N*_*v*_), the density-of-states effective mass is related to the band mass of an individual valley as $m^{*}=N_{v}^{3/2}m_{b}^{*}$ ,^53^ where *N*_*v*_ is almost unaffected by Yb in our case.^33^ As carrier mobility is inversely proportional to band mass,^54^ a large mobility is achieved due to Yb-substitution. A high PF of ∼10 μWcm^–1^K^–2^ at 623 K for Mg_0.98_Cu_0.02_Yb_0.8_Zn_1.2_Sb_2_ was successfully achieved. Synergetically, Cu doping and Yb alloying lead to a significant improvement in electrical transport through optimization of carrier concentration and mobility. Figure 4d shows the temperature-dependent total thermal conductivity (κ_tot_) for all the samples Mg_1.78–*y*_Cu_0.02_Yb_*y*_Zn_1.2_Sb_2_ (*y* = 0, 0.2, 0.4, 0.6, 0.8, and 1.0). At room temperature, κ_tot_ decreases with an increasing Yb content up to 0.4 and then increases further. The lowest κ_tot_ of ∼0.675 Wm^–1^K^–1^ is achieved at 673 K for Mg_1.18_Cu_0.02_Yb_0.6_Zn_1.2_Sb_2_. The reduction of κ_tot_ is mostly due to the reduction of the lattice thermal conductivity (κ_lat_). Figure 4e shows temperature-dependent κ_lat_ (κ_lat_ = κ_tot_ – κ_ele_) for all of the samples. At room temperature, the κ_lat_ decreases with Yb content until 0.4 and further increases with an increase in Yb because of the contribution from the electronic part. Near room temperature, the reduction in κ_lat_ is attributed to enhanced grain boundary scattering, as evidenced by the notably smaller grain sizes observed in Yb-alloyed samples compared to their Yb-free counterparts (see SI Figure S1). This microstructural refinement likely contributes to the observed increase in the grain boundary scattering. A similar observation of reduced grain size was also reported by Li et al. in their study.^16^ With an increase in temperature, the κ_lat_ decreases. The value of κ_lat_ was reduced to ∼0.37 Wm^–1^K^–1^ for *y* = 0.6 at 673 K. With an increase in temperature, scattering of high-frequency phonons by point defects plays an important role in reducing κ_lat_. In our system, the point defect scattering originates mostly from atomic mass fluctuation scattering, arising from the significant mass difference between Yb (173.05 g mol^–1^) and Mg (24.31 g mol^–1^) atoms.^44^ Similar findings in Yb-substituted *AB*_2_ *Sb*_2_ Zintl compounds have been reported earlier.^16,30,55^

The lattice thermal conductivity in crystalline solids is inherently governed by the material’s phonon dynamics, with the sound velocity (ν_*s*_) serving as a key descriptor of acoustic phonon transport. According to the Debye model, $\kappa_{\mathrm{lat}}=\frac{1}{3}C_{v}\nu_{s}^{2}\tau_{c}$ , where *C*_*v*_ is the phonon heat capacity and τ_c_ is the phonon relaxation time. The reduction in sound velocity, caused by softened elastic moduli and lattice expansion, leads directly to lower κ_lat_. The thermophysical properties of Mg_1.78–*y*_Cu_0.02_Yb_*y*_Zn_1.2_Sb_2_ (*y* = 0, 0.2, 0.4, 0.6, 0.8, and 1.0) demonstrate a complex interplay between mass, size, and lattice dynamics in determining lattice thermal conductivity. To get more insights into phonon dynamics, we measured the longitudinal (ν_l_) and transverse (ν_t_) sound velocities of the material at room temperature using a sing-around ultrasonic pulse technique (see the Experimental Details and SI for details). From these data, the average acoustic sound velocity (ν_s_) and corresponding elastic moduli, shear modulus (*G*), bulk modulus (*B*), and Young’s modulus (*E*), were derived and employed for phonon transport analysis (see SI for details).

The atomic mass and ionic radius of A-site cations (Mg and Yb) substantially impact the speed of sound, elastic moduli, and Grüneisen parameters (γ) in Zintl compounds. The measured (ν_l_) and (ν_t_) for Mg_1.78–*y*_Cu_0.02_Yb_*y*_Zn_1.2_Sb_2_ fall in the range of ν_l_ = 3913 m/s and ν_t_ = 2045 m/s. These values are in close alignment with other *p*-type Zintl phases such as Eu_2_ZnSb_2_ (ν_l_ = 3170 m/s and ν_t_ = 1900 m/s),^56^ YbMg_2_Sb_2_ (ν_l_ = 3100 m/s),^57^ and even favorably with SnSe (ν_l_ = 3100),^58^ however, they are quite lower than non-Zintl classical high thermal conductivity materials such as Si or Mg_2_Si (ν_l_ = 6200 m/s),^59^ indicating pronounced lattice softening in the presently studied material. The corresponding κ_lat_ reaches an ultra-low value of ∼0.37 Wm^–1^K^–1^ at 673 K that is even lower than the phonon-glass Zintl phases like Eu_2_ZnSb_2_ (κ_lat_ = 0.5–0.6 Wm^–1^K^–1^ above the room temperature).^56^ These moderate sound velocities indicate a relatively soft lattice, as confirmed by the reduced shear and bulk moduli compared to larger cation variants like CaMg_2_Sb_2_.^60^ Notably, this elastic softening stems from the small ionic radius of Mg (160.2 pm for Mg and 193.9 pm for Yb),^44^ which is undersized for the octahedrally coordinated site and leads to highly distorted Mg–Sb bonds. The substitution of Mg with larger Yb ions partly stabilizes the structure yet retains significant lattice disorder. Unlike Zintl compounds with lighter A-site cations (CaMg_2_Sb_2_), which exhibit higher elastic moduli and sound velocities, Zintl phases with mixed cation occupancy, such as Eu_2_ZnSb_2_ and Yb_14_MnSb_11_, experience strong mass fluctuation scattering and consequently achieve lower κ_lat_ mirroring the present finding of ultralow κ_lat_ of 0.37 Wm^–1^K^–1^ at 673 K for Mg_1.18_Cu_0.02_Yb_0.6_Zn_1.2_Sb_2_. Dependence of κ_lat_ on ν_s_ for various TE materials is shown in Figure S4 for comparison.

Mechanistically, the reduction in elastic moduli, evidenced by the shear modulus (*G*) and bulk modulus (*K*), stems from weakened and anisotropic bonding caused by size mismatch and atomic mass diversity. This leads to enhanced anharmonicity, an elevated Grüneisen parameter (γ > 1.8), and a broadened spectrometer of phonon scatterers, both static (mass fluctuations) and dynamic (lattice distortions). Thus, mass and size engineering, via targeted alloying at the A-site, represents a robust, designable route for pushing κ_*lat*_ toward its minimum in TE Zintl phases, leveraging combined effects of lowered sound velocity, softened lattice, and maximized phonon–phonon and defect scattering, firmly supported by both experimental results and advanced theoretical models to understand the route of mass fluctuation scattering.

For evaluating the thermal conductivity reduction, we use the model developed by Callaway and Klemens,^61,62^ later used by Abeles^63^ for solid solutions. If we consider only point defects and Umklapp scattering in an alloyed system,

$$\frac{\kappa_{p}}{\kappa_{\mathrm{lat}}}=\frac{\tan^{-1}\;u}{u}$$ 1

Here, κ_p_ and κ_lat_ are the lattice thermal conductivities of the host (no disorder) and defected (with disorder) system. And the term *u* in eq 1 is the disorder scaling parameter, which is defined by,

$$u={\left(\frac{\pi^{2}\theta_{D}\Omega }{h\nu^{2}}\kappa_{\mathrm{lat}}\Gamma_{\exp}\right)}^{1/2}$$ 2

where Γ_exp_ in eq 2 is the experimental disorder parameter, which gives the information about point defect scattering, which is also written as Γ_exp_ = Γ_M_ + Γ_S_, where Γ_M_ and Γ_S_ are disorder terms for mass fluctuation and strain (size) fluctuation, respectively. The values of Γ_M_ and Γ_S_ are calculated using the equation described elsewhere (see SI for details). The fitting parameters for Abeles’ equation are given in SI, here only the mass and strain fluctuation terms from the Yb atom replacing the *Mg1*-site are considered. The calculation for Γ_M_ and Γ_S_ shows that the mass disorder term dominates over the strain. In Mg_1.78–*y*_Cu_0.02_Yb_*y*_Zn_1.2_Sb_2_, the primary mechanism responsible for lowering κ_lat_ is mass fluctuation scattering resulting from the partial substitution of lighter Mg with heavier Yb atoms, i.e., also reflected in the calculation of Γ_exp_.

The temperature dependence of *zT* is shown in Figure 4f. An improved PF and reduced thermal conductivity via Yb alloying showed an enhanced *zT* of ∼0.95 at 673 K for the Mg_1.18_Cu_0.02_Yb_0.6_Zn_1.2_Sb_2_. This value is comparable to previously reported literature *zT* values.^16,30–33^ The comparison of the *zT* value for the present work and previously reported literature is given in Figure S6 (see SI).

## Development of Electrical Contacts

The composition Mg_1.18_Cu_0.02_Yb_0.6_Zn_1.2_Sb_2_ (referred to as ‘MCYZS-0.6’ hereafter) with the highest *zT* value was selected for device development. Low-resistance, stable electrical contacts are crucial for maximizing the performance of TE devices. In this study, initial trials were performed using a one-step sintering method with Fe and Ni contacts, as these materials have previously demonstrated low contact resistance when used with similar *p*-type TE materials.^30,31,67^ However, one-step sintering resulted in severe cracks on MCYZS-0.6 due to its poor mechanical strength and mismatched coefficient of thermal expansion (CTE) with the Fe and Ni (CTE of Mg_1.7_Na_0.01_Zn_1_Yb_0.25_Sb_2_ ∼ 17.5 × 10^–6^ K^–1^, CTE of Fe ∼ 12 × 10^–6^ K^–1^, CTE of Ni ∼ 13 × 10^–6^ K^–1^ at 325 K.^32^). Similar issues were reported by Hu et al.^32^ while making Fe contact with *p*-type Na_0.01_Mg_1.7_Zn_1_Yb_0.25_Sb_2_ by one-step sintering. In addition to Fe and Ni, a Cu70–Ni30 alloy (cupronickel, abbreviated as ‘CuNi’ hereafter) was employed as the contact material for MCYZS-0.6 due to its proven ability to form stable, low-resistance junctions on *n*-type Mg_3_(Sb,Bi)_2_-based materials^38^ and matching CTE (CTE of CuNi is ∼16.2 × 10^–6^ K^–1^ at 293–573 K^68^) with MCYZS-0.6. Based on the previous reports^30,64^ on CTE (∼20 × 10^–6^ K^–1^) of compositions similar to those studied here, we expected the present material to exhibit good thermal and mechanical compatibility with cupronickel contact. However, excessive melting was observed during the CuNi one-step sintering at 923 K, due to the formation of low-temperature eutectic phases.

To alleviate excessive melting issues, a two-step sintering process was implemented to fabricate CuNi contacts on MCYZS-0.6. In the first step, the MCYZS-0.6 powder was sintered at 923 K to form dense TE pellets, as schematically shown in Figure 5a. During the second step, the TE pellet was sandwiched between the contact layer powders and sintered at 773 K under 80 MPa pressure. This approach successfully prevented excessive melting issues. CuNi contacts exhibited crack-free, uniform interfaces without delamination, as observed in the micrograph in Figure 5b. EDS elemental mapping on the CuNi/MCYZS-0.6 interface (Figure 5b) did not show any interfacial reaction layer formation.

**Figure 5:**
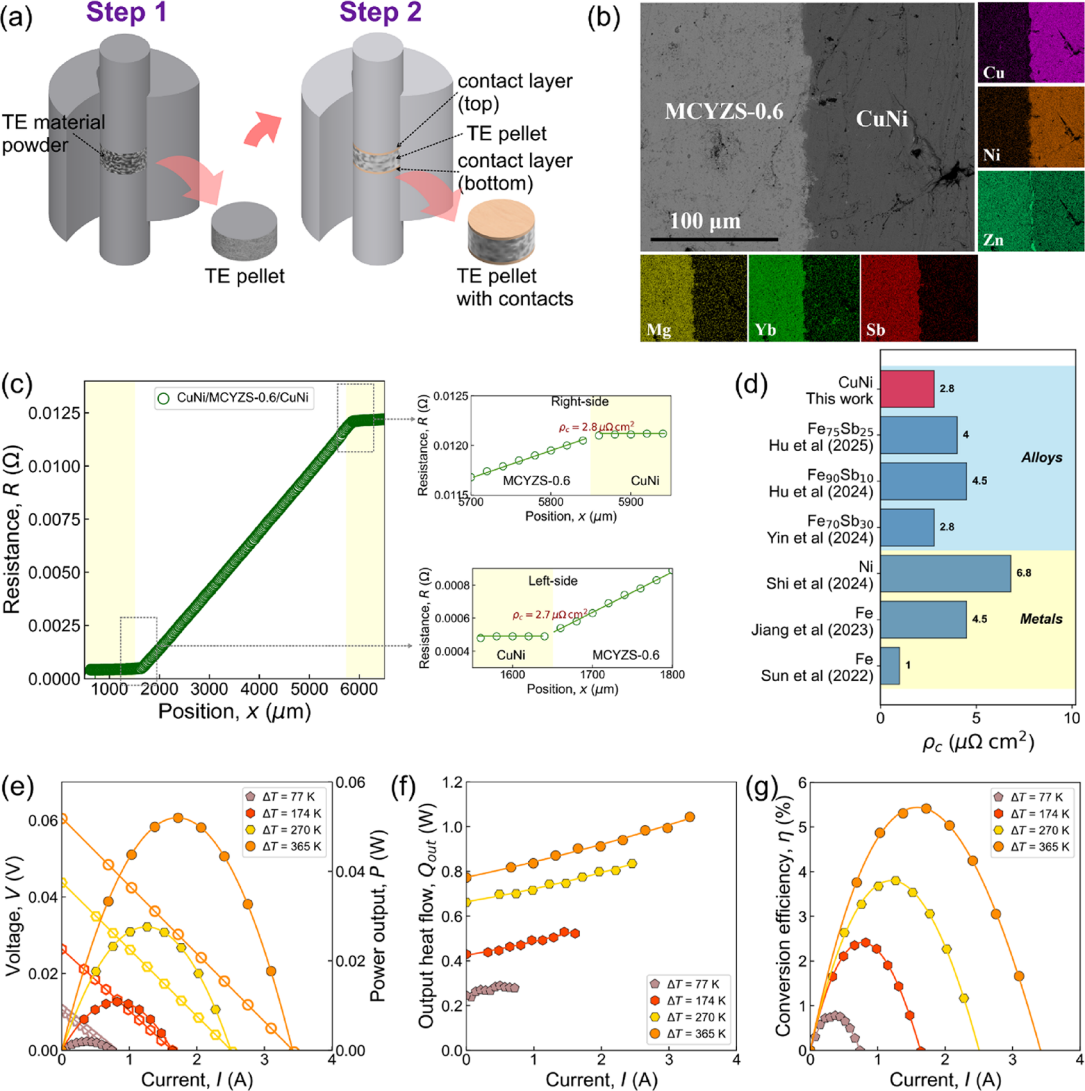
(a) Schematic of the two-step sintering for Ni and CuNi contact fabrication on MCYZS-0.6 samples; (b) SEM micrograph and EDS mapping across the CuNi/MCYZS-0.6 interface; (c) resistance profiling across the CuNi/MCYZS-0.6/CuNi samples at room temperature, the contact resistance estimation of CuNi/MCYZS-0.6 is given in the zoomed portion; (d) comparison of ρ_*c*_ with recent reported *p*-type Zintl materials; power generation characteristics of the CuNi/MCYZS-0.6/CuNi single leg. (e) V–I and P–I characteristics; (f) Q_out_-I characteristics; (g) η-I characteristics.

The specific contact resistivity (ρ_c_) across the CuNi/MCYZS-0.6/CuNi contacts at room temperature was calculated from the linear resistance profiling given in Figure 5c. Several line scans were recorded, and the arithmetic mean is reported. A relatively lower value of ∼2.7 μΩcm^2^ at the left side and ∼2.8 μΩcm^2^ at the right side was obtained for the CuNi/MCYZS-0.6/CuNi sample, shown in the zoomed region in Figure 5c. This value is one of the lowest ρ_c_ values reported for the *p*-type Zintl materials so far, as shown in Figure 5d. A few recent studies reported ∼4.5 μΩcm^2^ with Fe contacts on *p*-type Mg_1.98_Ag_0.02_ZnSb_2_ and ∼4 μΩcm^2^ with Fe_75_Sb_25_ contacts on Na_0.01_Mg_1.7_Zn_1_Yb_0.25_Sb_2_, both by one-step sintering.^31,32^ Also, the room temperature σ, calculated from the slope of the resistance profile at the TE materials region, was found to be ∼432 S/cm for the CuNi/MCYZS-0.6. A similar room temperature σ value was observed for the bare MCYZS-0.6 sample (Figure 4a), indicating that CuNi contacts did not affect the TE properties during the two-step contacting process. The power generation characteristics of the CuNi/MCYZS-0.6/CuNi TE leg were carried out at different Δ*T* values, keeping the cold side in the 293–300 K range. Figure 5e–g shows the *V*-*I*, *P*-*I*, *Q*_out_-*I*, and η-*I* characteristics of the CuNi/MCYZS-0.6/CuNi TE single leg. The open-circuit voltage (*V*_oc_), obtained from the intercept on the voltage axis, increased from 10.6 mV at Δ*T* = 77 K to 60.5 mV at Δ*T* = 365 K. The internal resistance (*R*_in_) of the TE leg, calculated from the slope of the *V*–*I* curve, also increased from 14.7 to 17.7 mΩ when Δ*T* increased from 77 to 365 K, due to lower σ at high temperature. A maximum power output (*P*_max_) of 52 mW is obtained for the CuNi/MCYZS-0.6/CuNi TE leg at Δ*T* = 365 K. The maximum conversion efficiency (η_max_) obtained was ∼5.5% at Δ*T* = 365 K. To verify the stability of the contact layers, we performed the aging test by annealing the TE leg for 3 days at 673 K. The microstructure analysis revealed that there are no secondary phases present at the interfaces of the CuNi/MCYZS-0.6 TE leg. Also, the specific contact resistivity is not varied significantly (before ∼2.7 μΩcm^2^ and after aging ∼4.1 μΩcm^2^, see SI Figure S8), indicating the good thermal stability of the suggested contact layer for *p*-type Mg_3_Sb_2_-based material. Although the single-leg efficiency is lower than that of *n*-type Mg_3_Sb_2_-based materials reported at Δ*T* = 365 K,^69,70^ integrating our *p*-type material with high-performance *n*-type counterparts opens the possibility of developing fully Mg_3_Sb_2_-based TE devices.

## Device Fabrication and Performance Testing

In order to understand the full potential of the developed *p*-type Mg_1.78–*y*_Cu_0.02_Yb_*y*_Zn_1.2_Sb_2_ material, the optimized MCYZS-0.6 composition is paired with a reported *n*-type Mg_3_Sb_1.5_Bi_0.49_Te_0.01_Cu_0.01_ material^8^ to fabricate a fully Mg_3_Sb_2_-based TE device. Figure 6a shows a schematic of the components used for fabricating the two-pair device. CuNi contacts were chosen for both *p*-type and *n*-type TE materials, fabricated by two-step sintering.^38^ Employing the same contact material for both *p-* and *n*-type TE materials can mitigate thermal stress and enhance versatility in device fabrication. For maximum efficiency gain, an optimized cross-sectional area

**Figure 6:**
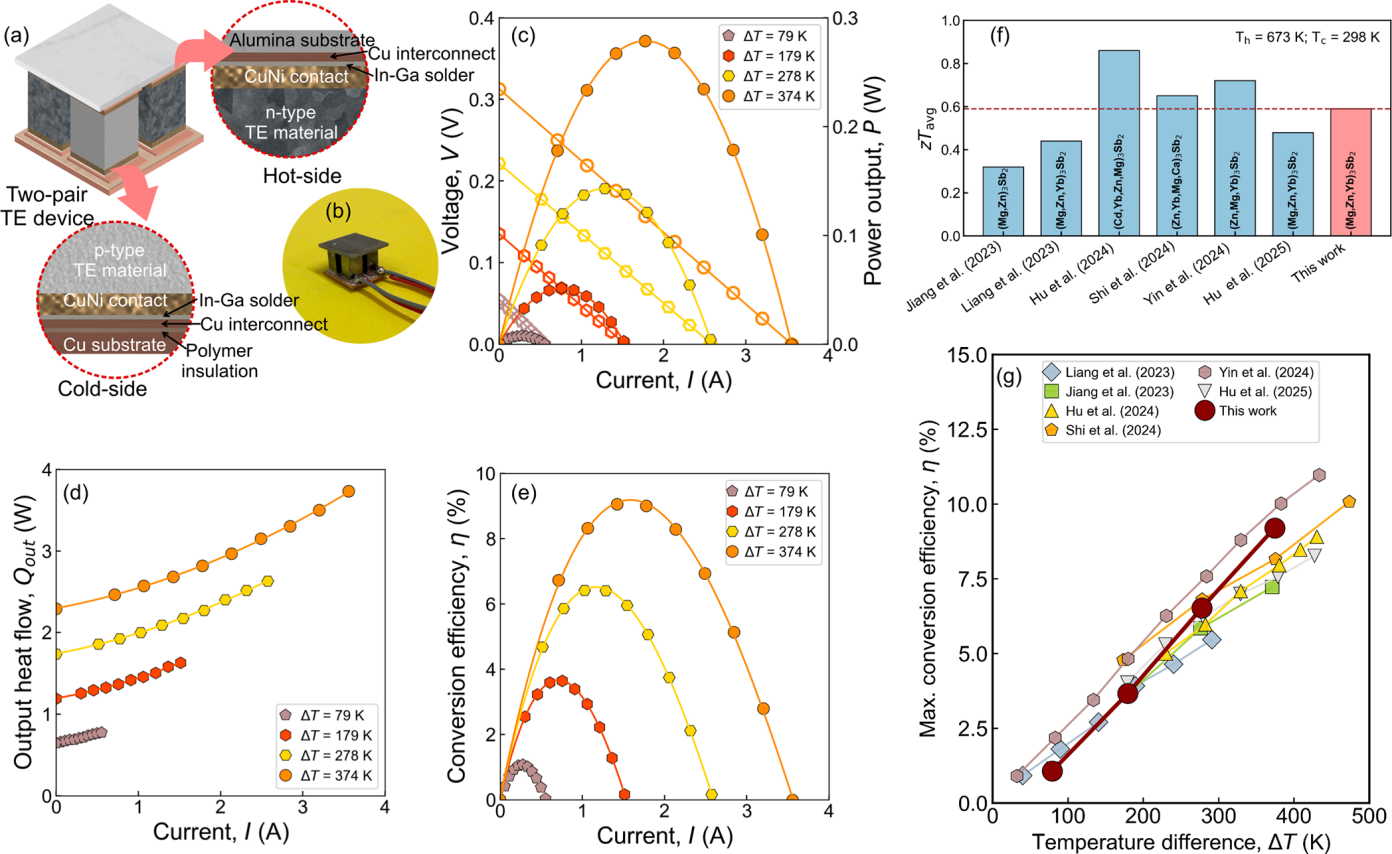
(a) Internal architecture of the fabricated two-pair device; (b) photo image of the two-pair device; (c) *V*-*I* and *P*-*I* characteristics of the two-pair device; (d) *Q*_out_-*I* characteristics and (e) η-I characteristics of the two-pair device; (f) average *zT* of the reported *p*-type Mg_3_Sb_2_-based Zintl compounds at Δ*T* = 375 K;^30–32,64–66^ (g) Maximum conversion efficiency (η_max_) of the reported fully Mg_3_Sb_2_-based TE devices made with *p*-type materials mentioned in Figure 5f and *n*-type Mg_3_(Sb,Bi)_2_ at different Δ*T.* ^30–32,64–66^

ratio (*A*_*n*_ */A*_*p*_) of ∼ 1:1 is adapted for preparing the TE legs, obtained from average TE properties, according to eqn [Disp-formula eq3]:^4^

$$\frac{A_{n}}{A_{p}}=\sqrt{\frac{\sigma_{p,\mathrm{av}}\kappa_{p,\mathrm{av}}}{\sigma_{n,\mathrm{av}}\kappa_{n,\mathrm{av}}}}$$ 3

The average electrical and thermal conductivity of *p*-type and *n-*type materials (σ_*p*,av_, κ_*p*,av_, σ_*n*,av_, and κ_*n*,av_) were calculated using a constant properties model (CPM).^71^ Accordingly, a two-pair prototype device of 10 × 10 mm^2^ size was successfully fabricated as shown in Figure 6b. The details of the substrates and component dimensions were provided in the Experimental Details section.

Figure 6c shows the *V*-*I* and *P*-*I* characteristics of the two-pair device. The *V*_oc_ increases from 55 to 312 mV when the Δ*T* increases from 79 to 374 K, as the Seebeck coefficient of both *n*-type and *p*-type TE materials shows an increasing trend with *T*. The device’s *R*_in_ was maintained nearly the same (∼87–92 mΩ) at all Δ*T* from 79 to 374 K. As a result, a *P*_max_ of ∼278 mW is obtained at Δ*T* = 374 K, which corresponds to a maximum power density (*ω*_max_) of 0.28 W cm^–2^. Figure 6d shows the measured heat flow at different Δ*T* and found increasing as a function of *I* at each measured Δ*T* due to the generated heat from Peltier, Joule, and Thomson effects.^39^ Figure 6e shows the variation of η with *I* for different Δ*T*. With increasing Δ*T*, η_max_ shows an increasing trend and obtains a maximum value of ∼9.2% at Δ*T* = 374 K. The higher η_max_ comes primarily from the high average figure of merit (*zT*_avg_) of the *p*-type Mg_1.18_Cu_0.02_Yb_0.6_Zn_1.2_Sb_2_ (0.59 at Δ*T* = 375 K, Figure 6f) and *n*-type Mg_3_Sb_1.5_Bi_0.49_Te_0.01_Cu_0.01_ (1.2 at Δ*T* = 375 K) and its low-resistance electrical contacts using CuNi. Moreover, we compared our results with those of the recently reported Mg_3_Sb_2_-based TE devices, as shown in Figure 6g. The obtained η_max_ is on par with the literature in the Δ*T* = 374 K range when the hot-side temperature is 673 K. We performed a thermal cycling test of the developed TE device to ensure stable performance for assessing the practical applicability. In this study, we performed 10 repeated thermal cycling tests at a temperature difference Δ*T* = 374 K and realized that the electrical output power and conversion efficiency are reproducible within ±5% of the measured data (see SI Figure S9). These results indicate the good thermal stability of the TE device with good output performance over extended operation. The high η_max_ of recently reported all-Mg_3_Sb_2_-based devices underscores their suitability for medium-temperature heat recovery applications. Apart from this, TE devices with the same parent material counterparts offer better reliability. This study reports on the fabrication of a fully Mg_3_Sb_2_-based module, along with the development of a high performance *p*-type material.

## Conclusions

In conclusion, the synergistic effects of Cu doping and Yb alloying in the Mg_3_Sb_2_ system effectively optimize the carrier concentration and reduce lattice thermal conductivity, leading to an enhanced TE performance with a peak *zT* of 0.95 at 673 K for Mg_1.18_Cu_0.02_Yb_0.6_Zn_1.2_Sb_2_. Moreover, the two-pair all-Mg_3_Sb_2_ TE device was fabricated using CuNi as the common interface layer for *p*-type Mg_1.18_Cu_0.02_Yb_0.6_Zn_1.2_Sb_2_ and *n*-type Mg_3_(Sb,Bi)_2_, demonstrating a high energy conversion efficiency of ∼9.2% under Δ*T* of 374 K. Notably, the adoption of the same contact interface layer for both *p-* and *n*-legs proved highly effective, yielding a low specific contact resistivity of ∼2.76 μΩcm^2^ for the *p*-type leg and ensuring superior interface quality and electrical stability. Overall, this work advances the performance of *p*-type Mg_3_Sb_2_-based materials and validates the feasibility of all-Mg_3_Sb_2_ TE devices for efficient and sustainable mid-temperature waste heat recovery applications.

## Supplementary Material



## Notes

Temperature-dependent heat capacity (*C*_*p*_) calculation, calculation of temperature-dependent Lorentz number, lattice parameter of Mg_1.8–*x*_Cu_*x*_Zn_1.2_Sb_2_ (*x* = 0, 0.01, 0.02, 0.03), scanning electron micrographs of fracture surface of Mg_1.78_Cu_0.02_Zn_1.2_Sb_2_ and Mg_1.18_Cu_0.02_Zn_1.2_Yb_0.6_Sb_2_, energy dispersive spectroscopy (EDS) analysis of Mg_1.18_Cu_0.02_Zn_1.2_Yb_0.6_Sb_2_, lattice parameter versus Yb composition plots, scanning electron micrographs of fracture surface of Mg_1.78_Cu_0.02_Zn_1.2_Sb_2_ and Mg_1.18_Cu_0.02_Zn_1.2_Yb_0.6_Sb_2_, density of states effective mass calculation, disorder scaling parameter calculation, sound velocity analyses, lattice thermal conductivity versus sound velocity plot for various materials, lattice and electronic thermal conductivity dependence on the temperature plot for Mg_1.78‑*y*_Cu_0.02_Zn_1.2_Yb_*y*_Sb_2_ (*y* = 0, 0.2, 0.4, 0.6, 0.8, 1.0), comparison of maximum *zT* reported in previous literature, COMSOL analysis of two-pair module, resistance scan plot for the CuNi/MCYZS-0.6/CuNi TE leg which is annealed for 3 days at 673 K, specific contact resistivity value is also mentioned, and power output and conversion efficiency of the CuNi/MCYZS-0.6/CuNi module for 10 cycles (PDF)

## References

[1] Bell L. E. (2008). Cooling,
Heating, Generating Power, and Recovering Waste Heat with Thermoelectric
Systems. Science (1979).

[2] Hendricks T., Caillat T., Mori T. (2022). Keynote Review
of Latest Advances
in Thermoelectric Generation Materials, Devices, and Technologies
2022. Energies.

[3] Liu Z., Tian B., Li Y., Guo Z., Zhang Z., Luo Z., Zhao L., Lin Q., Lee C., Jiang Z. (2023). Evolution
of Thermoelectric Generators: From Application to Hybridization. Small.

[4] Ioffe, A. F. Semiconductor Thermoelements, and Thermoelectric Cooling; Infosearch: London, 1957.

[5] Tamaki H., Sato H. K., Kanno T. (2016). Isotropic Conduction
Network and
Defect Chemistry in Mg_3_Sb_2_-based Layered Zintl
Compounds with High Thermoelectric Performance. Adv. Mater..

[6] Yang J., Li G., Zhu H., Chen N., Lu T., Gao J., Guo L., Xiang J., Sun P., Yao Y., Yang R., Zhao H. (2022). Next-Generation Thermoelectric Cooling Modules Based on High-Performance
Mg_3_(Bi,Sb)_2_ Material. Joule.

[7] Lei J., Wuliji H., Zhao K., Wei T. R., Xu Q., Li P., Qiu P., Shi X. (2021). Efficient Lanthanide Gd Doping Promoting
the Thermoelectric Performance of Mg_3_Sb_2_-Based
Materials. J. Mater. Chem. A.

[8] Liu Z., Sato N., Gao W., Yubuta K., Kawamoto N., Mitome M., Kurashima K., Owada Y., Nagase K., Lee C. H., Yi J., Tsuchiya K., Mori T. (2021). Demonstration
of Ultrahigh Thermoelectric Efficiency of 7.3% in Mg_3_Sb_2_/MgAgSb Module for Low-Temperature Energy Harvesting. Joule.

[9] Shi X., Zhao T., Zhang X., Sun C., Chen Z., Lin S., Li W., Gu H., Pei Y. (2019). Extraordinary *n*-type
Mg_3_SbBi Thermoelectrics Enabled by Yttrium
Doping. Adv. Mater..

[10] Ying P., He R., Mao J., Zhang Q., Reith H., Sui J., Ren Z., Nielsch K., Schierning G. (2021). Towards Tellurium-free Thermoelectric
Modules for Power Generation From Low-grade Heat. Nat. Commun..

[11] Peng W., Petretto G., Rignanese G. M., Hautier G., Zevalkink A. (2018). An Unlikely
Route to Low Lattice Thermal Conductivity: Small Atoms in a Simple
Layered Structure. Joule.

[12] Bano S., Chetty R., Babu J., Mori T. (2024). Mori, Mg_3_Sb_2_-based Material Mg_3_Sb_2_ and Devices
Rivaling Bismuth Telluride for Thermoelectric Power Generation and
Cooling. Device.

[13] Shuai J., Wang Y., Kim H. S., Liu Z., Sun J., Chen S., Sui J., Ren Z. (2015). Thermoelectric Properties
of Na-Doped Zintl Compound: Mg_3–*x*
_Na_
*x*
_Sb_2_. Acta Mater..

[14] Niu Y., Yang C., Zhou T., Pan Y., Song J., Jiang J., Wang C. (2020). Enhanced Average Thermoelectric Figure
of Merit of *p*-type Zintl Phase Mg_3_Sb_2_ via Zn Vacancy Tuning and Hole Doping. ACS Appl. Mater. Interfaces..

[15] Huang L., Liu T., Mo X., Yuan G., Wang R., Liu H., Lei X., Zhang Q., Ren Z. (2021). Thermoelectric Performance Improvement
of P-type Mg_3_Sb_2_-Based Materials by Zn and Ag
Co Doping. Mater. Today Phys..

[16] Li J., Liu K., Ma X., Yang Z., Yi L., Mao J., Zhang Q. (2024). Improvement
of the Thermoelectric Properties of *p*-type Mg_3_Sb_2_ by Mg-site Double Substitution. Inorg. Chem..

[17] Cho H., Back S. Y., Sato N., Liu Z., Gao W., Wang L., Nguyen H. D., Kawamoto N., Mori T. (2024). Outstanding
Room-temperature Thermoelectric Performance of *n*-type
Mg_3_Sb_2_Based Compounds Through Synergistically
Combined Band Engineering Approaches. Adv. Funct.
Mater..

[18] Song L., Zhang J., Iversen B. B. (2017). Simultaneous Improvement
of Power
Factor and Thermal Conductivity via Ag Doping in *p*-type Mg_3_Sb_2_ Thermoelectric Materials. J. Mater. Chem. A.

[19] Tiadi M., Trivedi V., Kumar S., Jain P. K., Yadav S. K., Gopalan R., Satapathy D. K., Battabyal M. (2023). Enhanced Thermoelectric
Efficiency in *p*-type Mg_3_Sb_2_: Role of Monovalent Atoms Codoping at Mg Sites. ACS Appl.Mater. Interfaces..

[20] Xie L., Yang J., Liu Z., Qu N., Dong X., Zhu J., Shi W., Wu H., Peng G., Guo F., Zhang Y., Cai W., Wu H., Zhu H., Zhao H., Liu Z., Sui J. (2023). Highly Efficient Thermoelectric
Cooling Performance of Ultrafine-grained and Nanoporous Materials. Mater. Today.

[21] Li A., Wang L., Li J., Mori T. (2024). Global Softening to
Manipulate Sound Velocity for Reliable High-performance MgAgSb Thermoelectrics. Energy Environ. Sci..

[22] Li A., Wang L., Wu X., Li J., Wang X., Wu G., Hu Z., Mori T. (2025). Semiconductor-Metal Transition Powers
High Efficiency MgAgSb Thermoelectrics. Sci.
Adv..

[23] Liu Z., Gao W., Oshima H., Nagase K., Lee C. H., Mori T. (2022). Maximizing
the Performance of *n*-type Mg_3_Sb_2_ Based Materials for Room-temperature power Generation and Thermoelectric
Cooling. Nat. Commun..

[24] Wu X., Lin Y., Han Z., Li H., Liu C., Wang Y., Zhang P., Zhu K., Jiang F., Huang J., Fan H., Cheng F., Ge B., Liu W. (2022). Interface and Surface
Engineering Realized High Efficiency of 13% and Improved Thermal Stability
in Mg_3_Sb_1.5_Bi_0.5_-based Thermoelectric
Generation Devices. Adv. Energy Mater..

[25] Sun Y., Zhu Y., Wu H., Qu N., Xie L., Zhu J., Liu Z., Zhang Q., Cai W., Guo F., Sui J. (2024). Rational Design
From Materials to Devices Enables an Efficiency of 10.5% Based On
Thermoelectric (Bi,Sb)_2_Te_3_ and Mg_3_(Bi,Sb)_2_ For Power Generation. Energy
Environ. Sci..

[26] Xu C., Liang Z., Shang H., Wang D., Wang H., Ding F., Mao J., Ren Z. (2021). Scalable Synthesis
of *n*-type Mg_3_Sb_2–*x*
_Bi_
*x*
_ for Thermoelectric Applications. Mater. Today Phys..

[27] Fu Y., Zhang Q., Hu Z., Jiang M., Huang A., Ai X., Wan S., Reith H., Wang L., Nielsch K., Jiang W. (2022). Mg_3_(Bi,Sb)_2_-based Thermoelectric Modules for
Efficient and Reliable Waste-heat Utilization Up To 750 K. Energy Environ. Sci..

[28] Bu Z., Zhang X., Hu Y., Chen Z., Lin S., Li W., Xiao C., Pei Y. (2022). A Record Thermoelectric Efficiency
in Tellurium-free Modules For Low-grade Waste heat recovery. Nat. Commun..

[29] Xu C., Liang Z., Ren W., Song S., Zhang F., Ren Z. (2022). Realizing High Energy
Conversion Efficiency in a Novel Segmented
Mg_3_(Sb,Bi)_2_/Cubic-GeTe Thermoelectric Module
for Power Generation. Adv. Energy Mater..

[30] Liang Z., Xu C., Song S., Shi X., Ren W., Ren Z. (2023). Enhanced Thermoelectric
Performance of *p*-type Mg_3_Sb_2_ for Reliable and Low-cost all-Mg_3_Sb_2_-based
Thermoelectric low Grade Heat Recovery. Adv.
Funct. Mater..

[31] Jiang M., Fu Y., Zhang Q., Hu Z., Huang A., Wang S., Wang L., Jiang W. (2023). High-efficiency
and Reliable Same-parent
Thermoelectric Modules Using Mg_3_Sb_2_-based Compounds. Natl. Sci. Rev..

[32] Hu J., Sun Y., Wu H., Yu Z., Zhu J., Guo F., Liu Z., Cai W., Sui J. (2025). All-Mg_3_Sb_2_-Based
Module for Thermoelectric Power Generation. Adv. Funct. Mater..

[33] Lei J., Wuliji H., Ren Q., Hao X., Dong H., Chen H., Wei T. R., Zhang J., Qiu P., Zhao K., Shi X. (2024). Exceptional Thermoelectric Performance
in AB_2_Sb_2_-type Zintl phases Through Band Shaping. Energy Environ. Sci..

[34] Liu C., Zhang Z., Peng Y., Li F., Miao L., Nishibori E., Chetty R., Bai X., Si R., Gao J., Wang X., Zhu Y., Wang N., Wei H., Mori T. (2023). Charge transfer engineering to achieve extraordinary power generation
in GeTe-based thermoelectric materials. Sci.
Adv..

[35] Rodríguez-Carvajal J. (1993). Recent Advances
in Magnetic Structure Determination by Neutron Powder Diffraction. Physica B Condens. Matter.

[36] Agne M. T., Imasato K., Anand S., Lee K., Bux S. K., Zevalkink A., Rettie A. J. E., Chung D. Y., Kanatzidis M. G., Snyder G. J. (2018). Heat Capacity of Mg_3_Sb_2_, Mg_3_Bi_2_, and Their Alloys at High Temperature. Mater. Today Phys..

[37] Alleno E., Bérardan D., Byl C., Candolfi C., Daou R., Decourt R., Guilmeau E., Hébert S., Hejtmanek J., Lenoir B., Masschelein P., Ohorodnichuk V., Pollet M., Populoh S., Ravot D., Rouleau O., Soulier M. (2015). Invited Article: A round Robin Test
of the Uncertainty on the Measurement of the Thermoelectric Dimensionless
Figure of Merit of Co_0.97_Ni_0.03_Sb_3_. Rev. Sci. Instrum..

[38] Chetty R., Babu J., Mori T. (2024). Improving Thermoelectric Conversion
Efficiency of Mg_3_(Sb,Bi)_2_-Based TE Materials
via Interface Contact Layer Optimization. ACS
Appl. Energy Mater..

[39] Chetty R., Babu J., Mori T. (2024). Best Practices for Evaluating the
Performance of Thermoelectric Devices. Joule.

[40] Löhberg K. (1934). Zur Kenntnis
Der Ersetzbarkeit von Zink Durch Magnesium Und Umgekehrt. Zeitschrift für Physikalische Chemie.

[41] Ren Z., Shuai J., Mao J., Zhu Q., Song S., Ni Y., Chen S. (2018). Significantly
Enhanced Thermoelectric Properties of *p*-type Mg_3_Sb_2_ via Co-doping of Na
and Zn. Acta Mater..

[42] Hu J., Zhu J., Guo F., Qin H., Liu Y., Zhang Q., Liu Z., Cai W., Sui J. (2022). Electronic Orbital Alignment and
Hierarchical Phonon Scattering Enabling High Thermoelectric Performance
P-Type Mg 3 Sb 2 Zintl Compounds. Research.

[43] Tang X., Zhang B., Zhang X., Wang S., Lu X., Han G., Wang G., Zhou X. (2020). Enhancing the Thermoelectric
Performance
of *p*-type Mg_3_Sb_2_ via Codoping
of Li and Cd. ACS Appl. Mater. Interfaces..

[44] Haynes, W. M. CRC Handbook of Chemistry and Physics, CRC Press, 2016.

[45] Song L., Zhang J., Iversen B. B. (2019). Thermal
stability of p-type Ag-doped
Mg 3 Sb 2 thermoelectric materials investigated by powder X-ray diffraction. Phys. Chem. Chem. Phys..

[46] Chen J., Xue W., Chen C., Li H., Cai C., Zhang Q., Wang Y. (2021). All-Scale Hierarchical Structure Contributing to Ultralow Thermal
Conductivity of Zintl Phase CaAg_0.2_Zn_0.4_Sb. Adv. Sci..

[47] Shuai J., Geng H., Lan Y., Zhu Z., Wang C., Liu Z., Bao J., Chu C. W., Sui J., Ren Z. (2016). Higher Thermoelectric
Performance of Zintl phases (Eu_0.5_Yb_0.5_)_1–*x*
_Ca_
*x*
_Mg_2_Bi_2_ by Band engineering and Strain Fluctuation. Proc. Natl. Acad. Sci. U. S. A..

[48] Snyder G. J., Pereyra A., Gurunathan R. (2022). Effective Mass from Seebeck Coefficient. Adv. Funct. Mater..

[49] Zhang J., Song L., Madsen G. K. H., Fischer K. F. F., Zhang W., Shi X., Iversen B. B. (2016). Designing High-Performance Layered Thermoelectric Materials
Through Orbital Engineering. Nat. Commun..

[50] Liu W., Hu J., Zhang S., Deng M., Han C. G., Liu Y. (2017). New Trends,
Strategies and Opportunities in Thermoelectric Materials: A Perspective. Mater. Today Phys..

[51] Zhang J., Song L., Iversen B. B. (2019). Insights Into the
Design of Thermoelectric
Mg_3_Sb_2_ and its Analogs by Combining Theory and
Experiment. npj Comput. Mater..

[52] Zhang X., Luo H., Cao X., Han G., Wu H., Zhang Y., Zhang B., Wang G., Zhou X. (2025). Achieving
Excellent
Thermoelectric Performance in *p*-type Mg_3_Sb_2_-Based Zintl Materials Via Synergistic Band Engineering
and Entropy Engineering. Acta Mater..

[53] Snyder G. J., Toberer E. S. (2008). Complex Thermoelectric
Materials. Nat. Mater..

[54] Huang Y., Lei J., Chen H., Zhou Z., Dong H., Yang S., Gao H., Wei T. R., Zhao K., Shi X. (2023). Intrinsically High
Thermoelectric Performance in Near-Room-Temperature _α_-MgAgSb Materials. Acta Mater..

[55] Zhang Z., Yao H., Wang Q., Xue W., Wang Y., Yin L., Wang X., Li X., Chen C., Sui J., Lin X., Chen Y., Liu X., Mao J., Xie G., Zhang Q. (2022). Achieving High Thermoelectric
Performance in Severely Distorted YbCd_2_Sb_2_. Adv. Funct. Mater..

[56] Chen C., Feng Z., Yao H., Cao F., Lei B. H., Wang Y., Chen Y., Singh D. J., Zhang Q. (2021). Intrinsic
Nanostructure Induced Ultralow Thermal Conductivity Yields Enhanced
Thermoelectric Performance in Zintl Phase Eu_2_ZnSb_2_. Nat. Commun..

[57] Mehrotra K., Novitskii A., Mori T. (2025). Understanding the Prospects of the
Thermoelectric Performance of The YbMg_2_(Bi,Sb)_2_ Zintl Phase. Chem. Mater..

[58] Xiao Y., Chang C., Pei Y., Wu D., Peng K., Zhou X., Gong S., He J., Zhang Y., Zeng Z., Zhao L.-D. (2016). Origin of Low Thermal
Conductivity
in SnSe. Phys. Rev. B.

[59] Madelung, O. ; Rössler, U. ; Schulz, M. , Magnesium Silicide (Mg2Si) Sound Velocities, Elastic Moduli. In Non-Tetrahedrally Bonded Elements and Binary Compounds I; Eds.; Landolt-Börnstein Group III Condensed Matter; Springer-Verlag: Berlin/Heidelberg, 1998; Vol. 41C, pp 1–4 10.1007/10681727_105.

[60] Wood M., Aydemir U., Ohno S., Snyder G. J. (2018). Observation
of Valence
Band Crossing: The Thermoelectric Properties of CaZn_2_Sb_2_ – CaMg_2_Sb_2_ Solid Solution. J. Mater. Chem. A.

[61] Callaway J., von Baeyer H. C. (1960). von Baeyer, Effect Of Point Imperfections on Lattice
Thermal Conductivity. Phys. Rev..

[62] Klemens P. G. (1955). The Scattering
of Low-Frequency Lattice Waves by Static Imperfections. Proc. Phys. Soc. A.

[63] Abeles B. (1963). Lattice Thermal
Conductivity of Disordered Semiconductor Alloys at High Temperatures. Phys. Rev..

[64] Hu J., Sun Y., Shi W., Wu H., Zhu J., Cheng J., Jiao L., Jiang X., Xie L., Qu N., Li F., Yu Z., Zhang Q., Liu Z., Guo F., Cai W., Sui J. (2024). Realizing Ultrahigh
Conversion Efficiency of 9.0% in
YbCd_2_Sb_2_/Mg_3_Sb_2_ Zintl
Module for Thermoelectric Power generation. Adv. Mater..

[65] Shi X., Song S., Gao G., Ren Z. (2024). Global Band Convergence
Design for High-Performance Thermoelectric Power Generation in Zintls. Science.

[66] Yin L., Li X., Bao X., Cheng J., Chen C., Zhang Z., Liu X., Cao F., Mao J., Zhang Q. (2024). CALPHAD Accelerated
Design of Advanced Full-Zintl Thermoelectric Device. Nat. Commun..

[67] Sun Y., Yin L., Zhang Z., He H., Chen C., Li S., Chen L., Jia J., Wang X., Sui J., Liu X., Mao J., Cao F., Zhang Q. (2022). Low Contact Resistivity
and Excellent Thermal Stability of *p*-type YbMg_0.8_Zn_1.2_Sb_2_/Fe-Sb Junction for Thermoelectric
Applications. Acta Mater..

[68] Davis, J. R. Copper and Copper Alloys; ASM International, 2001.

[69] Zhu Q., Song S., Zhu H., Ren Z. (2019). Realizing High Conversion
Efficiency of Mg_3_Sb_2_-Based Thermoelectric Materials. J. Power Source.

[70] Wang L., Zhang W., Back S. Y., Kawamoto N., Nguyen D. H., Mori T. (2024). High-Performance Mg_3_Sb_2_-Based Thermoelectrics
with Reduced Structural Disorder and Microstructure Evolution. Nat. Commun..

[71] Jayachandran B., Dasgupta T., Singh A. (2023). A Constant
Properties Model for the
Performance Estimation in Segmented Thermoelectric Generator Elements
and Its Experimental Validation Using an *n*-type Mg_2_Si_0.3_Sn_0.7_-Bi_2_Te_2.7_Se_0.3_ Segmented Leg. ACS Appl. Energy
Mater..

