# Supplementary material for: Synergistic Cu Doping and Yb Alloying Enhance Thermoelectric Performance of p‑Type Mg1.8Zn1.2Sb2‑Based Material toward High-Efficiency All-Mg3Sb2 Devices
Source: ACS Appl Mater Interfaces. 2026 Jan 30;18(7):11358–69. doi: 10.1021/acsami.5c22459 (PMC12954663; doi:10.1021/acsami.5c22459)
Supplement: Supplementary file 1 [file am5c22459_si_001.pdf]

Supporting Information

Synergistic Cu Doping and Yb Alloying Enhance Thermoelectric Performance of p-type $Mg_{1.8}Zn_{1.2}Sb_2$ based material toward High-Efficiency All-Mg_3Sb_2 Devices

Krushna Kumari Raut,[a,b] Raju Chetty,[b] Jayachandran Babu,[b] Andrei Novitskii,[b] Vikrant Trivedi,[b] and Takao Mori*,[a,b]

[a] *Graduate School of Pure and Applied Sciences, University of Tsukuba, 1-1-1 Tennodai, Tsukuba, Ibaraki 305-8573, Japan.*

[b] *Research Center for Materials Nanoarchitectonics (MANA), National Institute for Materials Science (NIMS), Tsukuba,1-1 Namiki, Ibaraki 305-0044, Japan.*

* E-mail: MORI.Takao@nims.go.jp

Temperature-dependent heat capacity (C_p) calculation

At high temperatures, it is essential to evaluate the C_p of Mg_3Sb_2-based materials, as enhanced lattice vibrations and thermal expansion significantly influence their values compared to those at room temperature. We have used the formula developed by Agne et al.[1] to calculate the temperature dependent C_p values,

$$C_p \ \left[\mathrm{J\ g^{-1}\ K^{-1}}\right] = \frac{3NR}{M_W}\left(1 + 1.3 \times 10^{-4}\, T - 4 \times 10^{3}\, T^{-2}\right) \tag{S1}$$

Where, 3NR= 124.71 $\mathrm{J\,mol^{-1}\,K^{-1}}$ and M_W is the molecular weight

Calculation of temperature-dependent Lorentz Number

The lattice contribution to thermal conductivity (κ_{lat}) was derived by subtracting the electronic component (κ_{ele}) from the total thermal conductivity (κ_{tot}). The electronic part of thermal conductivity (κ_{ele}=LσT) is calculated from the Weidemann-Franz relation using the temperature dependent Lorenz number, L(T). By using following equation (1) we have calculated the L(T) from the experimental Seebeck coefficient (α) values.[2]

$$L = 1.5 + \exp\left(-\frac{|\alpha|}{116}\right) \tag{S2}$$

This empirical formula is derived from original single parabolic band model(SPB) where L is in $10^{-8}\ \mathrm{W\,\Omega\,K^{-2}}$ and α is in μV/K are related through Fermi integrals and band parameters.

$$L = \left(\frac{k_B}{e}\right)^2 \frac{(1+\lambda)(3+\lambda)F_\lambda(\eta)F_{\lambda+2}(\eta) - \left[(2+\lambda)F_{\lambda+1}(\eta)\right]^2}{\left[(1+\lambda)F_\lambda(\eta)\right]^2} \tag{S3}$$

$$\alpha = \frac{k_B}{e}\left(\frac{(2+\lambda)F_{\lambda+1}(\eta)}{(1+\lambda)F_\lambda(\eta)} - \eta\right) \tag{S4}$$

Where, k_B is the Boltzmann's constant, λ is the scattering parameter, $F_{\lambda+2}(\eta)$ is the Reduced Fermi Energy, α is the Seebeck coefficient, e is the elementary charge.

Lattice parameter of $\mathbf{Mg_{1.8-x}Cu_xZn_{1.2}Sb_2}$ (x = 0, 0.01, 0.02, 0.03)

Table S1: Lattice parameter of $Mg_{1.8-x}Cu_xZn_{1.2}Sb_2$ (x = 0, 0.01, 0.02, 0.03) from Rietveld refinement

**Composition**	**Lattice parameter (a)**	**Lattice parameter (c)**
x = 0	4.423	7.202
x = 0.01	4.429	7.199
x = 0.02	4.427	7.198
x = 0.03	4.427	7.196

Scanning Electron Micrographs of fracture surface of $Mg_{1.78}Cu_{0.02}Zn_{1.2}Sb_2$ and $Mg_{1.18}Cu_{0.02}Zn_{1.2}Yb_{0.6}Sb_2$

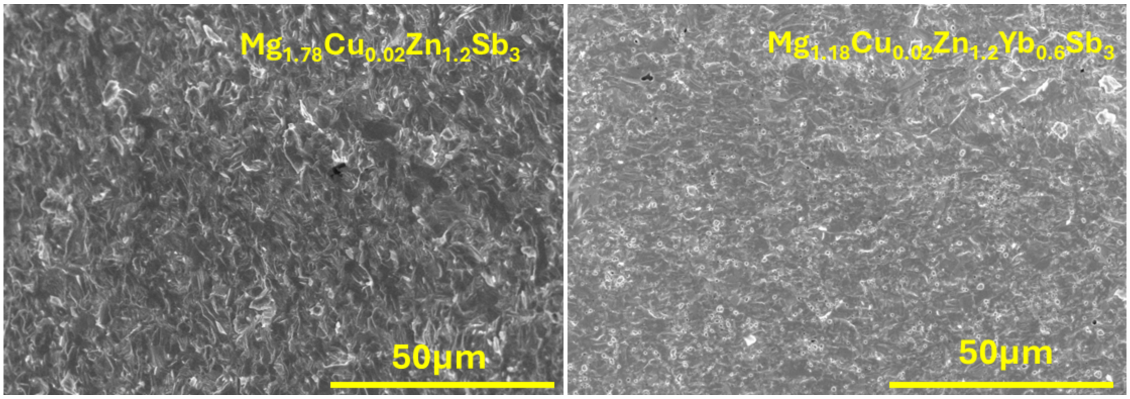

Figure S1: SEM micrographs of fracture surface of $Mg_{1.78}Cu_{0.02}Zn_{1.2}Sb_2$ and $Mg_{1.18}Cu_{0.02}Zn_{1.2}Yb_{0.6}Sb_2$ showing the smaller grains in Yb alloyed sample in compared to that of non-Yb sample

Table S2: Nominal and actual composition of $Mg_{1.18}Cu_{0.02}Yb_{0.6}Zn_{1.2}Sb_2$ by Energy Dispersive Spectroscopy (EDS)

**Elements**	**Nominal composition (at%)**	**Actual composition (at%)**
Mg	23.6	21.4
Cu	0.4	0.5
Zn	24.0	22.5
Sb	40.0	39.0
Yb	12.0	16.3

Lattice parameter verses Yb composition plots

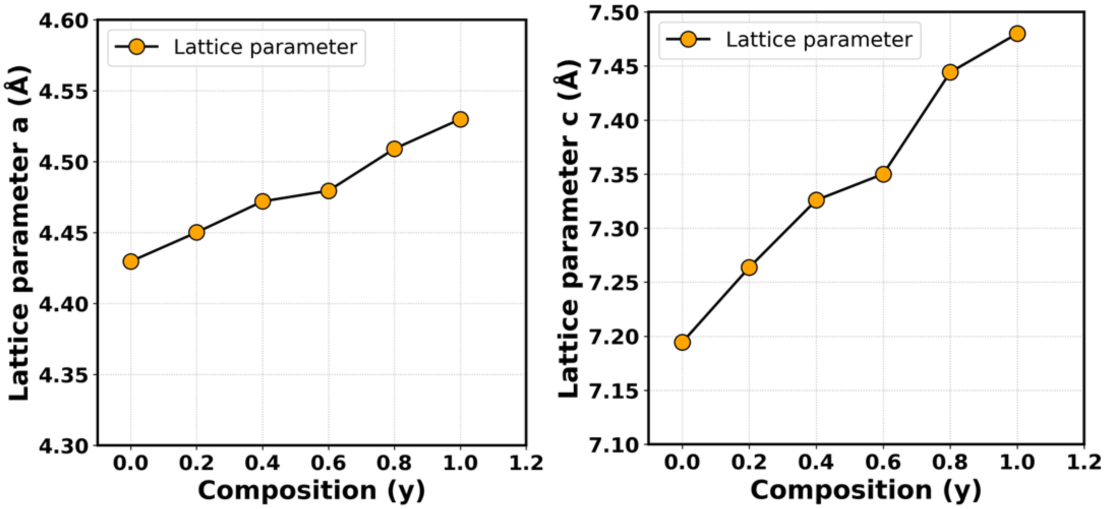

Figure S2: Variation of Lattice parameter with Yb content of $Mg_{1.78-y}Cu_{0.02}Yb_yZn_{1.2}Sb_2$ (y = 0, 0.2, 0.4, 0.6, 0.8 and 1.0)

Scanning Electron Micrographs of fracture surface of $Mg_{1.78}Cu_{0.02}Zn_{1.2}Sb_2$ and $Mg_{1.18}Cu_{0.02}Zn_{1.2}Yb_{0.6}Sb_2$

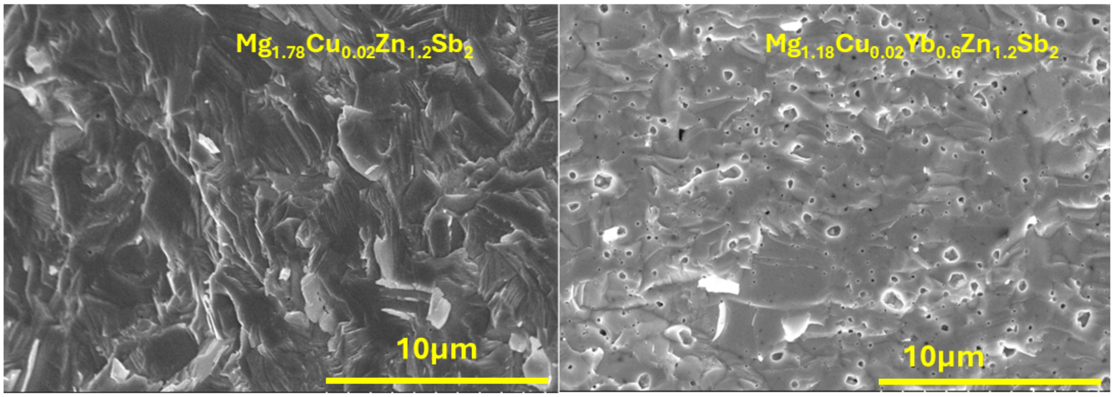

Figure S3: SEM micrographs of fracture surface of $Mg_{1.78}Cu_{0.02}Zn_{1.2}Sb_2$ and $Mg_{1.18}Cu_{0.02}Zn_{1.2}Yb_{0.6}Sb_2$.

Density of states effective mass calculation

We use equation provided by Snyder et al.[3] using Single parabolic Band (SPB) model, to calculate the density of states effective mass (m^*)

$$m^* = \frac{h^2}{2\pi k_B T} \left(\frac{3n_H}{\pi} \right)^{2/3} \exp \left(-\frac{2e|S|}{k_B} + 0.17 \right) \tag{S5}$$

It can be simplified as,

$$m^* = 0.924 \left(\frac{T}{300\,\mathrm{K}} \right)^{1/2} \left(\frac{n_H}{10^{19}\,\mathrm{cm}^{-3}} \right)^{1/3} \exp \left(\frac{2e|S|}{k_B} - 0.17 \right) \tag{S6}$$

where, h is Planck's constant, K_B is Boltzmann's constant, T is the absolute temperature, n_H is the Hall carrier concentration, S is the Seebeck coefficient and e is the elementary charge. Seebeck coefficient is closely related to the Fermi level position in semiconductors, representing the energy difference between the Fermi level and the band edge. When combined with the carrier concentration, which can be determined using the Hall effect, these measurements provide direct insight into the electronic density of states (DOS).

Disorder scaling parameter calculation

According to Abeles equation,[4]

$$u = \left(\frac{\pi^2 \theta_D \Omega}{h v^2} \kappa_{\mathrm{lat}} \Gamma_{\mathrm{exp}} \right)^{1/2} \tag{S7}$$

Where Γ_{exp} is the experimental disorder parameter which gives the clear information about point defect scattering which is also written as $\Gamma_{\mathrm{exp}} = \Gamma_{\mathrm{M}} + \Gamma_{\mathrm{S}}$, Γ_{M} and Γ_{S} are disorder terms for mass fluctuation and strain (size) fluctuation respectively. However, Γ value for individual elements in a compound is define by[5]),

$$\Gamma_M = \frac{\sum_{i=1}^{n} c_i \left(\frac{\overline{M_i}}{\overline{\overline{M}}} \right)^2 f_i^{k1} f_i^{k2} \left(\frac{M_i^1 - M_i^2}{\overline{M_i}} \right)^2}{\left(\sum_{i=1}^{n} c_i \right)} \tag{S8}$$

$$\Gamma_S = \frac{\sum_{i=1}^{n} c_i \left(\frac{\overline{M_i}}{\overline{\overline{M}}} \right)^2 \varepsilon_i}{\sum_{i=1}^{n} c_i} \sum_k f_i^k \left(1 - \frac{r_i^k}{\overline{r_i}} \right)^2 \tag{S9}$$

where, M is the total mass, c_i is the sublattice count, f_i^k is the filling fraction at site i of atom k, $\overline{M}$ is the average atomic mass of the compound/alloy, $\overline{M_i}$ and $\overline{r_i}$ are the avarage mass and avegare radius of the ith sublattice. ϵ_i is the phenomenological adjustable parameter and characterizes the anharmonicity inside the crystal lattice,[6] typically the value ranges from 10 to 100.[4] $\overline{M_i}$ and $\overline{M}$ are defined as,

$$\overline{M_i} = \sum_k f_i^k M_i^k \tag{S10}$$

$$\overline{M} = \frac{\sum_{i=1}^{n} c_i \overline{M}_i}{\sum_{i=1}^{n} c_i} \tag{S11}$$

For $Mg_{1.78-y}Cu_{0.02}Zn_{1.2}Yb_ySb_2$, the formula can be simplified as,

$$\overline{M} = \frac{1}{5} \left[M_1^{\mathrm{Mg}}(1-y) + M_1^{\mathrm{Yb}} y + 2 \times M_2^{\mathrm{Zn}}(1-x) + 2 \times M_2^{\mathrm{Zn}} x + M_3^{\mathrm{Sb}} \right] \tag{S12}$$

$$\Gamma_M = \frac{1}{5} \left[c_1 \left(\frac{\overline{M}_1}{\overline{M}} \right)^2 f_1^{\mathrm{Mg_1}} f_2^{\mathrm{Yb}} \left(\frac{M_1^{\mathrm{Mg_1}} - M_2^{\mathrm{Yb}}}{\overline{M}_1} \right)^2 + 0 + 0 \right] \tag{S13}$$

$$\Gamma_M = \frac{1}{5}\left[y(1-y)\left(\frac{M_1^{\mathrm{Mg_1}} - M_2^{\mathrm{Yb}}}{\overline{\overline{\overline{M}}}}\right)^2\right] \tag{S14}$$

Similarly, Γ_S can be written as,

$$\Gamma_S = \frac{1}{5}\left[y(1-y)\left(\frac{r_1^{\mathrm{Mg_1}} - r_2^{\mathrm{Yb}}}{\overline{\overline{\overline{M}}}}\right)^2 \varepsilon_i\right] \tag{S15}$$

Sound velocity analyses

In the Debye model,[7] the phonon density of states (DOS) scales as $g(\omega) \propto \frac{\omega^2}{v^3}$, so that each acoustic branch enters thermodynamic and transport integrals with a weight v^3. Accordingly, the average sound velocity (ν_s)is taken as the cubic-harmonic mean from measured longitudinal (ν_s) and transverse sound velocities (ν_t), using the following formula

$$v_s = \left(\frac{1}{3}\left[\frac{1}{v_l^3} + \frac{1}{v_t^3}\right]\right)^{-\frac{1}{3}} \tag{S16}$$

Debye temperature(θ_D^{Sv}) is defined as,

$$\theta_D^{Sv} = \frac{h}{k_B}\left(\frac{3N}{4\pi V_a}\right)^{\frac{1}{3}} v_s \tag{S17}$$

where V_a is the unit cell volume, N is the number of atoms in the unit cell, k_B is the Boltzmann constant and h represents the Planck constant. Grüneisen parameter(γ_a), can also be evaluated from the propagation velocities of acoustic waves as:

$$\gamma_a = \frac{3}{2}\left(\frac{3v_l^2 - 4v_t^2}{v_l^2 + 2v_t^2}\right) \tag{S18}$$

The Young's (E), bulk (K), and shear (G) moduli of the samples were calculated from measured sound velocities,

$$E = \frac{dv_t^2(3v_l^2 - 4v_t^2)}{v_l^2 - v_t^2} \tag{S19}$$

$$K = d\left(v_l^2 - \frac{4}{3}v_t^2\right) \tag{S20}$$

$$G = v_t^2 d \tag{S21}$$

d is the density of the samples measured through Archimedes' method with ethanol as the working fluid. The results are tabulated in Table S1.

Table S3: Room temperature elastic properties of $Mg_{1.78-y}Cu_{0.02}Yb_yZn_{1.2}Sb_2$ ($x = 0, 0.4, 1.0$)

**Composition**	**d (g/cm^3)**	ν_L **(m/s)**	ν_T **(m/s)**	ν_s **(m/s)**	θ_D **(K)**	γ	**u**	Γ_{exp}
$Mg_{1.78}Cu_{0.02}Zn_{1.2}Sb_2$	4.97	3913.04	2045.45	2288.24	235	1.85	–	–
$Mg_{1.38}Cu_{0.02}Yb_{0.4}Zn_{1.2}Sb_2$	5.24	3417.48	1944.75	2161.73	215	1.55	1.24	0.134
$Mg_{0.98}Cu_{0.02}Yb_{1.0}Zn_{1.2}Sb_2$	6.3	3646.47	2136.75	2370.8	235	1.48	–	–

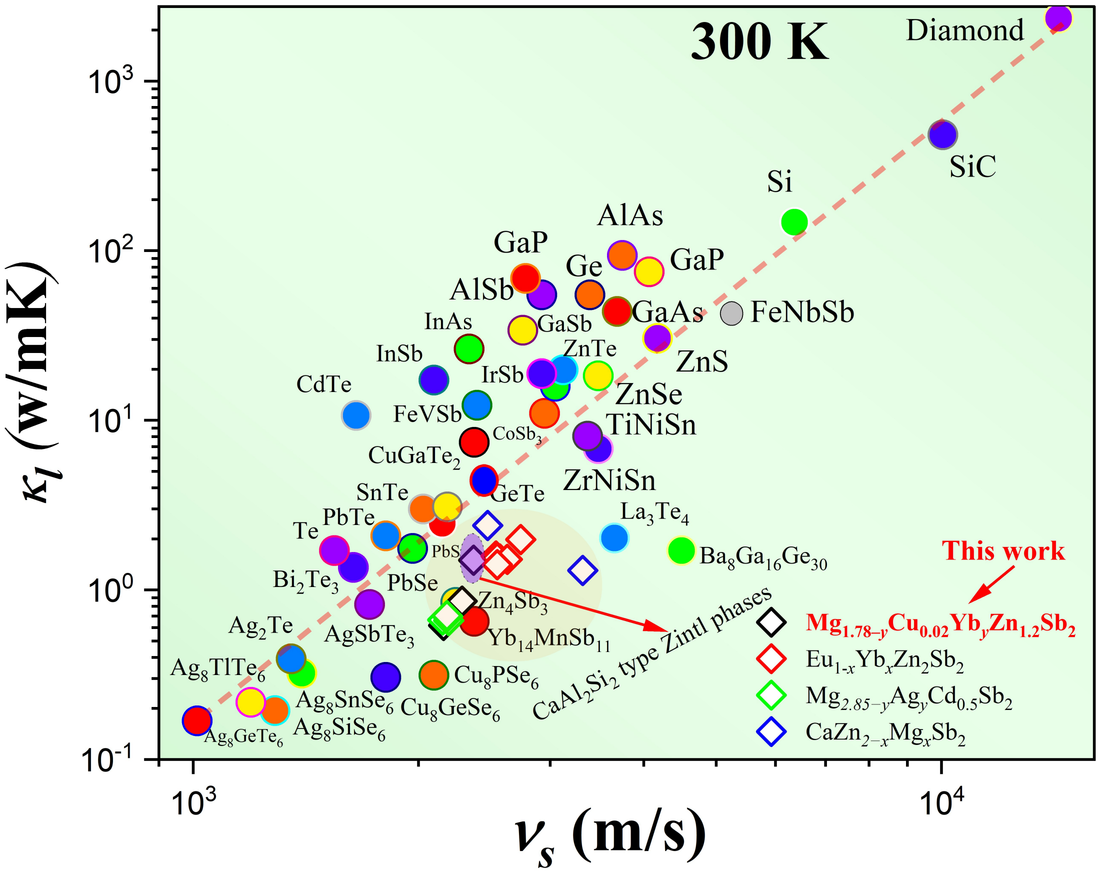

Figure S4: Lattice Thermal conductivity versus sound velocity plot for various materials [8,9]

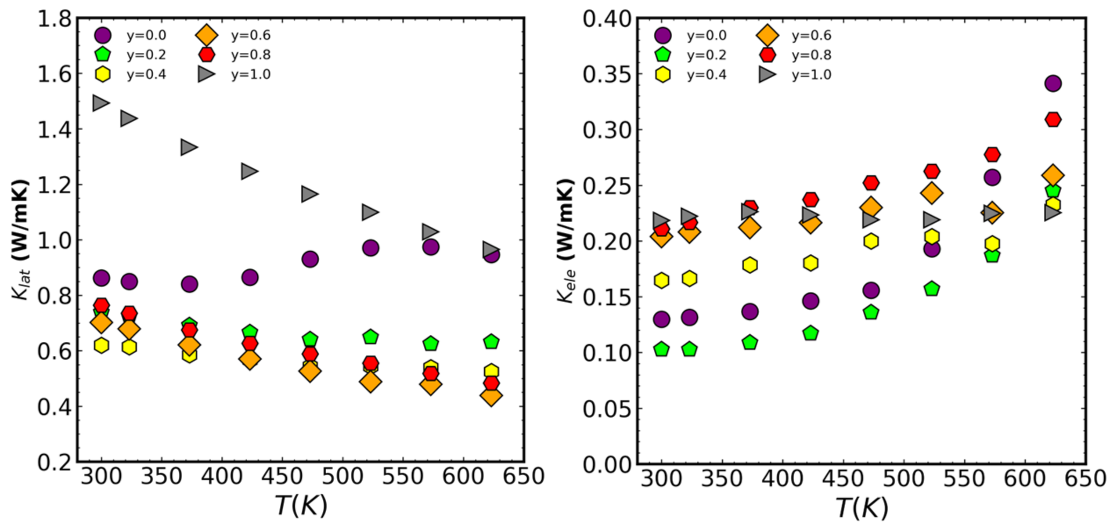

Figure S5: Lattice and electronic thermal conductivity dependence on temperature plot for $Mg_{1.78-y}Cu_{0.02}Zn_{1.2}Yb_ySb_2$ (y=0,0.2,0.4,0.6,0.8,1.0).

Comparision of maximum zT with previous literatures

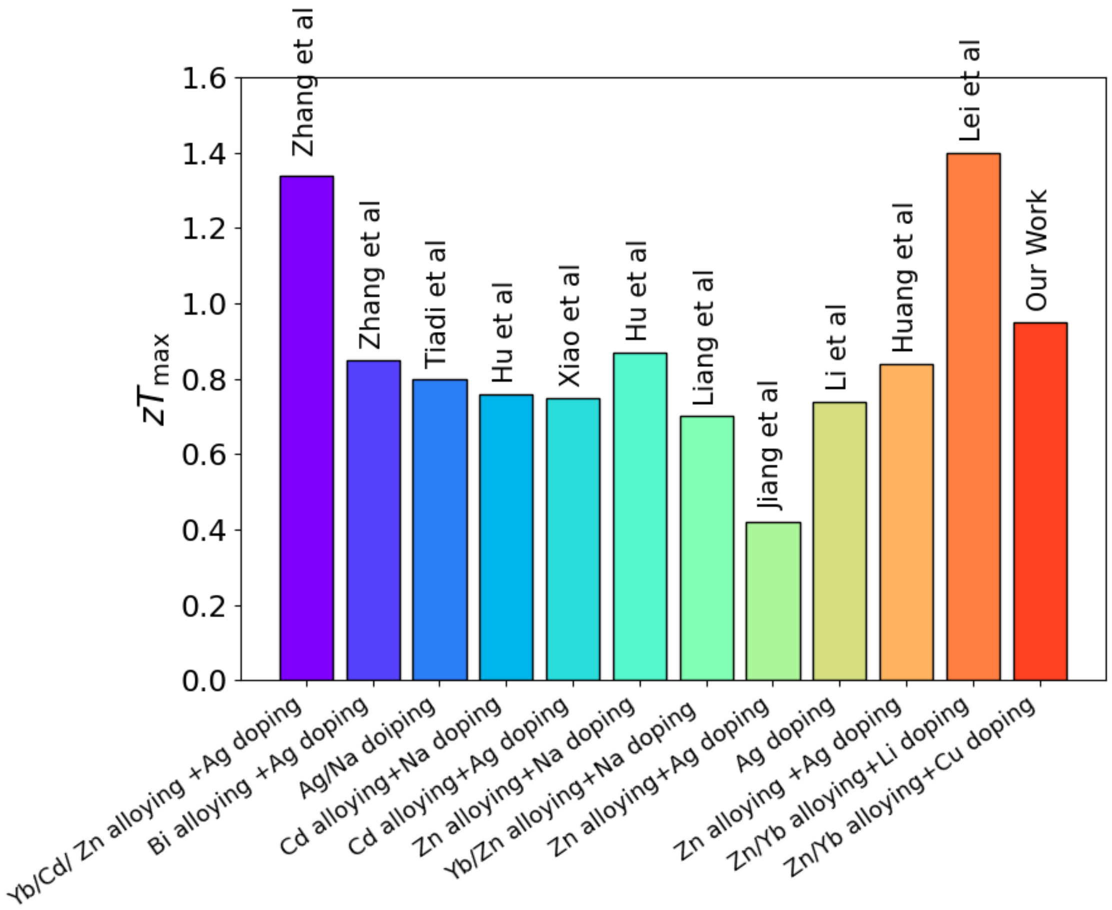

Figure S6: Comparison of maximum zT reported in previous literature[10–20]

COMSOL Analysis of two pair module

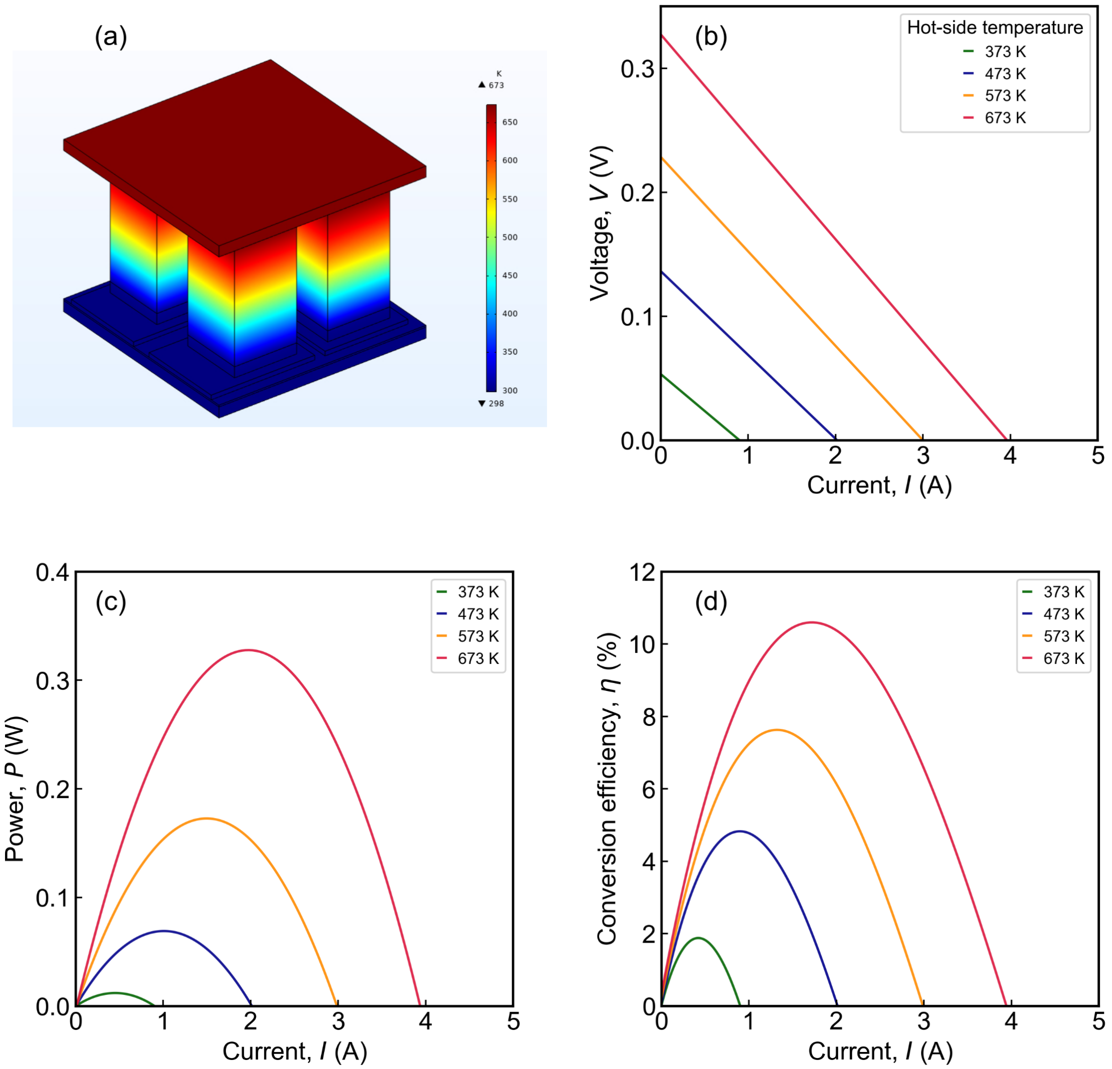

Figure S7: (a) Internal architecture of the simulated two-pair device (b) V-I characteristics of the two-pair device (c) P-I characteristics of the two-pair device (d) η-I characteristics of the two-pair device

Resistance scan plot for CuNi/MCYZS-0.6/CuNi after 3 days annealing at 673 K

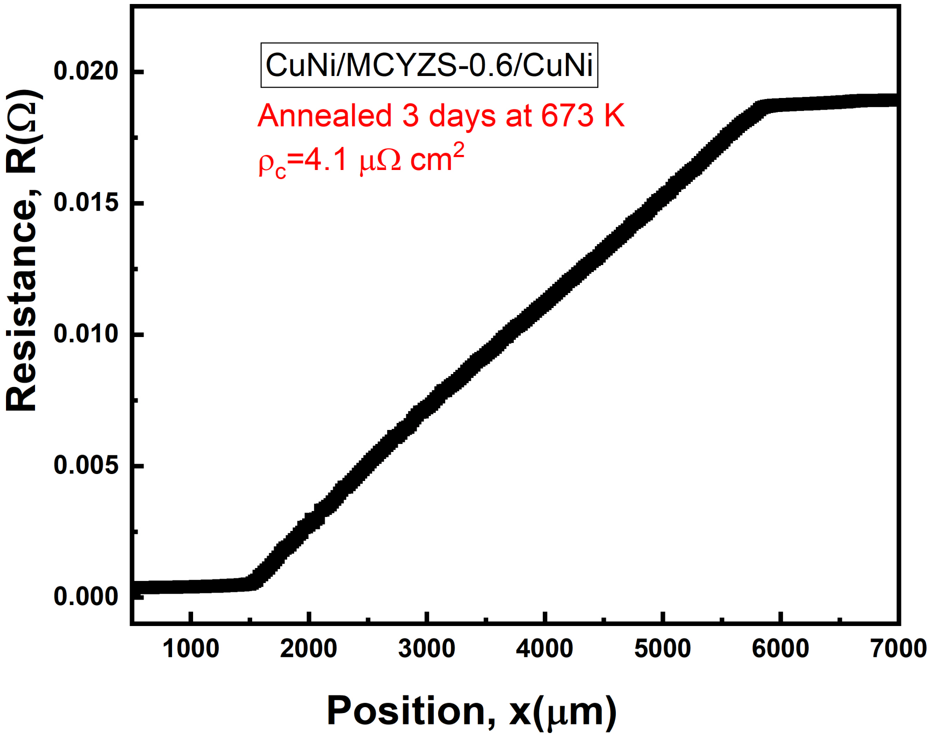

Figure S8: Resistance scan plot for CuNi/MCYZS-0.6/CuNi TE leg which is annealed for 3 days at 673 K, Specific contact resistivity value is also mentioned

Power output and Conversion efficiency of CuNi/MCYZS-0.6/CuNi module for 10 cycles

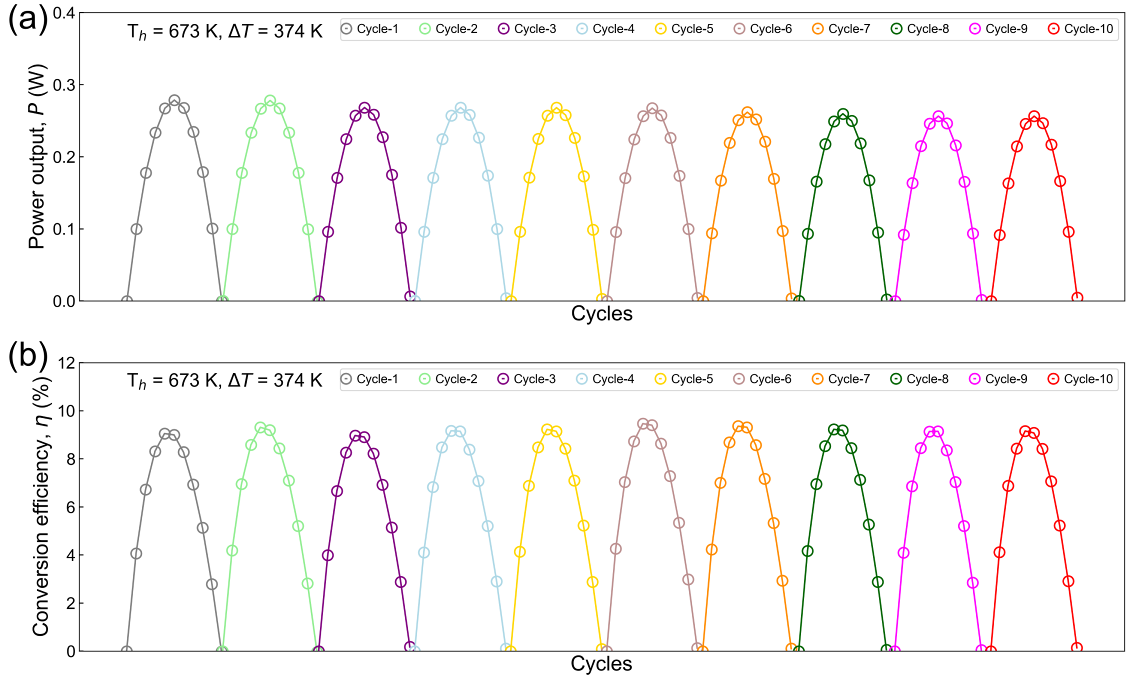

Figure S9: (a)Power output (b) Conversion efficiency ($\eta_{\max}$) of CuNi/MCYZS-0.6/CuNi module for 10 cycles

References

(1) Agne, M. T.; Imasato, K.; Anand, S.; Lee, K.; Bux, S. K.; Zevalkink, A.; Rettie, A. J.; Chung, D. Y.; Kanatzidis, M. G.; Snyder, G. J. Heat Capacity ofMg_3Sb_2, Mg_3Bi_2, and Their Alloys at High Temperature. *Mater. Today Phys.* **2018**, *6*, 83–88.

(2) Kim, H.-S.; Gibbs, Z. M.; Tang, Y.; Wang, H.; Snyder, G. J. Characterization of Lorenz number with Seebeck coefficient measurement. *APL mater.* **2015**, *3*.

(3) Snyder, G. J.; Pereyra, A.; Gurunathan, R. Effective Mass from Seebeck Coefficient. *Adv. Funct. Mater.* **2022**, *32*, 2112772.

(4) Abeles, B. Lattice Thermal Conductivity of Disordered Semiconductor Alloys at High Temperatures. *Phys. Rev.* **1963**, *131*, 1906.

(5) Yang, J.; Meisner, G.; Chen, L. Strain Field Fluctuation Effects on Lattice Thermal Conductivity of Zrnisn-Based Thermoelectric Compounds. *Appl. Phys. Lett.* **2004**, *85*, 1140–1142.

(6) Zhang, Z. et al. Achieving High Thermoelectric Performance in Severely Distorted $YbCd_2Sb_2$. *Adv. Funct. Mater.* **2022**, *32*, 2205215.

(7) Tritt, T. M. *Thermal Conductivity: Theory, Properties, and Applications*; Springer Science & Business Media, 2005.

(8) Li, W.; Lin, S.; Ge, B.; Yang, J.; Zhang, W.; Pei, Y. Low Sound Velocity Contributing to The High Thermoelectric Performance Of Ag_8SnSe_6. *Adv. Sci.* **2016**, *3*, 1600196.

(9) Magnesium silicide (Mg2Si) sound velocities, elastic moduli: Datasheet from Landolt-Börnstein - Group III Condensed Matter · Volume 41C: "Non-Tetrahedrally Bonded Elements and Binary Compounds I" in SpringerMaterials (https://doi.org/10.1007/10681727_105). https://materials.springer.com/lb/docs/sm_lbs_978-3-540-31360-1_105, Copyright 1998 Springer-Verlag Berlin Heidelberg.

(10) Zhang, X.; Luo, H.; Cao, X.; Han, G.; Wu, H.; Zhang, Y.; Zhang, B.; Wang, G.; Zhou, X. Achieving Excellent Thermoelectric Performance in *p*-type Mg_3Sb_2-Based Zintl Materials Via Synergistic Band Engineering and Entropy Engineering. *Acta Mater.* **2025**, *289*, 120933.

(11) Jiang, M.; Fu, Y.; Zhang, Q.; Hu, Z.; Huang, A.; Wang, S.; Wang, L.; Jiang, W. High-efficiency and Reliable Same-parent Thermoelectric Modules Using Mg_3Sb_2-based Compounds. *Natl. Sci. Rev.* **2023**, *10*, nwad095.

(12) Liang, Z.; Xu, C.; Song, S.; Shi, X.; Ren, W.; Ren, Z. Enhanced Thermoelectric Performance of *p*-type Mg_3Sb_2 for Reliable and Low-cost all-Mg_3Sb_2-based Thermoelectric low-Grade Heat Recovery. *Adv. Funct. Mater.* **2023**, *33*, 2210016.

(13) Li, X.; Yang, B.; Xie, H.; Zhong, H.; Feng, S.; Zhang, Y.; Ma, Y.; Zhang, J.; Su, H. Synergistic effects of Mg vacancy and Ag doping on thermoelectric transport properties of p-type Mg3Sb2. *Materials Research Bulletin* **2023**, *159*, 112106.

(14) Hu, J.; Zhu, J.; Guo, F.; Qin, H.; Liu, Y.; Zhang, Q.; Liu, Z.; Cai, W.; Sui, J. Electronic Orbital Alignment and Hierarchical Phonon Scattering Enabling High Thermoelectric Performance *p*-type Mg_3Sb_2 Zintl Compounds. *Research* **2022**, *2022*, 1–12.

(15) Xiao, S.; Peng, K.; Zhou, Z.; Wang, H.; Zheng, S.; Lu, X.; Han, G.; Wang, G.; Zhou, X. Realizing Cd and Ag codoping in p-type Mg3Sb2 toward high thermoelectric performance. *Journal of Magnesium and Alloys* **2023**, *11*, 2486–2494.

(16) Tiadi, M.; Trivedi, V.; Kumar, S.; Jain, P.; Yadav, S. K.; Gopalan, R.; Satapathy, D. K.; Battabyal, M. Enhanced Thermoelectric Efficiency in p-type Mg_3Sb_2: Role of Monovalent Atoms Codoping at Mg Sites. *ACS Appl.Mater. Interfaces.* **2023**, *15*, 20175–20190.

(17) Zhang, Y.-b.; Liang, J.-S.; Liu, C.; Zhou, Q.; Xu, Z.; Chen, H.-b.; Li, F.-c.; Peng, Y.; Miao, L. Enhancing thermoelectric performance in P-Type Mg3Sb2-based zintls through optimization of band gap structure and nanostructuring. *Journal of Materials Science & Technology* **2024**, *170*, 25–32.

(18) Huang, L.; Liu, T.; Mo, X.; Yuan, G.; Wang, R.; Liu, H.; Lei, X.; Zhang, Q.; Ren, Z. Thermoelectric Performance Improvement of P-type Mg_3Sb_2-Based Materials by Zn and Ag Co-Doping. *Mater. Today Phys.* **2021**, *21*, 100564.

(19) Hu, J.; Zhu, J.; Dong, X.; Guo, M.; Sun, Y.; Shi, W.; Zhu, Y.; Wu, H.; Guo, F.; Zhang, Y.-X.; others Breaking the minimum limit of thermal conductivity of Mg3Sb2 thermoelectric mediated by chemical alloying induced lattice instability. *Small* **2023**, *19*, 2301382.

(20) Lei, J.; Wuliji, H.; Ren, Q.; Hao, X.; Dong, H.; Chen, H.; Wei, T.-R.; Zhang, J.; Qiu, P.; Zhao, K.; Shi, X. Exceptional Thermoelectric Performance in AB_2Sb_2-type Zintl phases Through Band Shaping. *Energy Environ. Sci.* **2024**, *17*, 1416–1425.
